# Pharmacological Nephroprotection in Chronic Kidney Disease Patients with Type 2 Diabetes Mellitus—Clinical Practice Position Statement of the Polish Society of Nephrology

**DOI:** 10.3390/ijms252312941

**Published:** 2024-12-02

**Authors:** Marcin Adamczak, Ilona Kurnatowska, Beata Naumnik, Tomasz Stompór, Leszek Tylicki, Magdalena Krajewska

**Affiliations:** 1 Department of Nephrology, Transplantation and Internal Medicine, Medical University of Silesia, 40-027 Katowice, Poland; 2 Department of Internal Diseases and Transplant Nephrology, Medical University of Lodz, 90-153 Lodz, Poland; 3 1st Department of Nephrology, Transplantation and Internal Medicine with Dialysis Unit, Medical University of Bialystok, 15-540 Bialystok, Poland; bnaumnik@poczta.onet.pl; 4 Department of Nephrology, Hypertension and Internal Medicine, University of Warmia and Mazury in Olsztyn, 10-516 Olsztyn, Poland; stompin@mp.pl; 5 Department of Nephrology, Transplantology and Internal Medicine, Medical University of Gdansk, 80-952 Gdansk, Poland; 6 Department of Non-Surgical Clinical Sciences, Wroclaw University of Science and Technology, 50-370 Wroclaw, Poland; magda@softstar.pl

**Keywords:** diabetes mellitus type 2, chronic kidney disease, nephroprotection, blood pressure control, renin–angiotensin system, sodium–glucose co-transporter type 2 inhibitors, metabolic acidosis

## Abstract

Both chronic kidney disease (CKD) and type 2 diabetes (T2D) are modern epidemics worldwide and have become a severe public health problem. Chronic kidney disease progression in T2D patients is linked to the need for dialysis or kidney transplantation and represents the risk factor predisposing to serious cardiovascular complications. In recent years, important progress has occurred in nephroprotective pharmacotherapy in CKD patients with T2D. In the current position paper, we described a nephroprotective approach in CKD patients with T2D based on the five following pillars: effective antihyperglycemic treatment, SGLT2 inhibitor or semaglutide, antihypertensive therapy, use of RASi (ARB or ACEi), and in selected patients, finerenone, as well as sodium bicarbonate in patients with metabolic acidosis. We thought that the current statement is comprehensive and up-to-date and addresses multiple pathways of nephroprotection in patients with CKD and T2D.

## 1. Introduction

In recent years, there has been important progress in understanding the pathogenesis of kidney diseases and in treating patients with chronic kidney disease (CKD). To facilitate the implementation of the results of recent clinical trials into everyday clinical practice, the Polish Society of Nephrology decided to prepare clinical practice position statements covering different areas of clinical nephrology. After presenting the statements concerning pharmacological nephroprotection in non-diabetic CKD [1] and the use of intravascular contrast media in patients with impaired kidney function [2], the current statement refers to pharmacological nephroprotection in CKD with T2D patients.

### 1.1. Epidemiology of T2D and CKD Worldwide

In 2015, over 400 million people worldwide were affected by diabetes, and according to predictions, this number is expected to increase to 640 million by 2040. This is primarily due to the ageing of the population and lifestyle-related factors [3,4]. The incidence of diabetes in adults in Poland is 8% (of which 7.2% in men and 8.9% in women). Therefore, it is estimated that currently, over 2 million people in Poland suffer from diabetes (approximately 25% of whom remain undiagnosed). Projections suggest that in the next 15–20 years, the number of people with diabetes in Poland will double [5].

Chronic kidney disease is a frequent complication of both type 1 and type 2 diabetes (T2D), and globally, diabetic kidney disease is the leading cause of CKD and end-stage renal disease (ESRD) [6]. In patients with diabetes, a reduction in the glomerular filtration rate (GFR) and/or increased excretion of albumin in the urine for at least three months defines the diagnosis of CKD, as in the case of other patients. The development of CKD in the course of diabetes occurs in 40% of diabetic patients due to functional and structural kidney damage. Kidney injury in diabetes results from, among others, chronic hyperglycemia, the presence of arterial hypertension, and genetic factors. It should be emphasized that the actual prevalence of diabetic kidney disease and CKD attributed to diabetes is not precisely known, as kidney biopsy (the gold standard in diagnosing diabetic kidney disease) is rarely performed in patients with diabetes and CKD. However, it is estimated that in 30 to 60% of patients with T2D and CKD, mechanisms other than diabetes may also contribute to kidney injury. Diabetic kidney disease is a complex and heterogeneous condition with multiple overlapping etiological pathways, including changes in the hemodynamics of renal glomeruli, oxidative stress, inflammation, interstitial fibrosis, and atrophy of renal tubules. Owing to this complexity, it seems that even the best achievable glycemic control of diabetes through employing the use of antihyperglycemic drugs cannot effectively protect against CKD progression once kidney injury is already established (although it is essential to prevent the new-onset diabetic kidney injury defined as de novo albuminuria > 30 mg/g of creatinine) [7]. This will result in a huge burden on the healthcare system caused by an increased cardiovascular risk with a rise in morbidity and mortality from this cause, and what cannot be ignored is a significant increase in the number of people needing costly renal replacement therapy. For this reason, all possible actions must be taken to prevent the development and progression of kidney damage caused by diabetes. Educational activities and the promotion of proper therapeutic procedures fit into this trend.

### 1.2. Pathophysiology of Diabetic Renal Complications and Their Clinical Consequences

The pathogenesis of diabetic kidney injury is complex. Many pathways—such as metabolic, hemodynamic, inflammatory, and fibrotic—are triggered by hyperglycemia and ultimately lead to renal damage. Mediators of these pathways promote the development of structural and functional renal changes. Factors such as reactive oxygen species, inflammatory cytokines, and, for instance, TGFβ are shared across several pathways, resulting in significant interaction among them.

Elevated blood glucose levels activate pathways, including the hexosamine pathway, advanced glycation end-products (AGE) pathway, protein kinase C (PKC) pathway, and the polyol pathway [8].

Increased production of reactive oxygen species (ROS), along with elevated levels of mitogen-activated protein kinase (MAPK), signal transducer and activator of transcription proteins (JAK/STAT), and NF-κB, also occurs. This leads to inflammation and fibrosis [9].

Diabetes-related hemodynamic effects include elevated systemic and intraglomerular blood pressure, causing hyperfiltration that drives progressive albuminuria and a decline in GFR, influenced by circulating mediators such as angiotensin II (Ang II), thromboxane A2, and endothelin-1 (ET-1). A nitric oxide deficiency also increased cyclooxygenase-2 prostanoids and activation of the kallikrein-kinin system contributed to this process [10,11].

Tubular mechanisms also play a role in intraglomerular hypertension in diabetes; activation of glucose transport in the proximal tubule increases sodium reabsorption in the proximal nephron, while decreased sodium concentration triggers tubuloglomerular feedback [12].

Inflammatory processes are initiated during diabetes, resulting in the formation of inflammatory molecules such as tumor necrosis factor α (TNF-α), interleukin 1β (IL-1β), interleukin 6 (IL-6), and interleukin 18 (IL-18) [13,14,15]. Increased production and deposition of amyloid A protein also occur [16]. Complement system activation further influences DKD progression [17].

The familial occurrence of diabetic kidney disease suggests the role of genetic factors. Their interaction with epigenetic and environmental factors also impacts the initiation and progression of CKD in T2D patients [18].

Histone modifications are crucial in regulating gene expression patterns that contribute to disease progression, creating a molecular environment that promotes the activation of pro-inflammatory and pro-fibrotic pathways [19,20].

While hyperglycemia plays a crucial role, hyperinsulinemia, insulin resistance, and lipotoxicity also underly pathogenic mechanisms [21], ultimately, changes in the glomerular hemodynamics, inflammation, and fibrosis mediate kidney damage. However, the involvement of these mechanisms varies among individuals and throughout the natural course of diabetic kidney disease. It is important to note that in some patients, especially those with T2D, the most common type of this disease in adults (>90%), CKD may develop independently of diabetes. In these cases, diabetes is only a concurrent cause. The morbidity of CKD patients with T2D is a consequence of both macrovascular (atherosclerosis) and microvascular changes (e.g., retinopathy, nephropathy, neuropathy). In T2D, the diagnosis can be delayed because the onset of the disease is often insidious. Diabetes complications may be present at the time of diagnosis, and their severity increases over time. Chronic kidney disease associated with T2D, especially in advanced stages, poses a life-threatening condition. Its progression is linked to the need for dialysis or kidney transplantation. It represents the risk factor predisposing to serious cardiovascular (CV) complications, which are, in fact, the leading cause of death in these patients [22]. The risk of CV events and CV death increases exponentially with a decline in GFR and/or an increase in albuminuria, regardless of age, gender, or other risk factors [22,23]. The development and progression of CKD in T2D patients lead to a reduced quality of life, an increased frequency of disabilities, and premature deaths [24]. Healthcare costs are also significantly increased in individuals who develop CKD in the course of T2D [25].

### 1.3. The Main Goals of CKD in T2D Treatment

Due to all these factors, CKD in T2D patients is a severe public health problem. The treatment goals are to preserve kidney function, slow CKD progression, reduce ESRD risk, and decrease cardiovascular disease (CVD) risk. Effective therapy aimed at slowing the CKD progression and extending life expectancy should be holistic and involve medications with various mechanisms of action. The management of patients with CKD and T2D is similar in principle to that applied in all individuals with diabetes, but it includes certain specific recommendations. First, intensive blood pressure (BP) control is crucial in patients with CKD and T2D. Effective antihypertensive treatment is mandatory because BP control has been proven to slow the progression of CKD, prevent CVD, and reduce mortality. In most patients, the treatment of hypertension requires a combination of antihypertensive drugs from different groups. The initial treatment typically includes renin-angiotensin system (RAS) blockade, angiotensin receptor blockers (ARBs), or, in the case of ARB intolerance, angiotensin-converting enzyme inhibitors (ACEIs) adjusted to the maximum tolerated doses. These nephroprotective measures have been used in patients with CKD and T2D over the past 20 years [26]. Glycemic control for patients with CKD and T2D is defined as a hemoglobin A1c blood concentration of 7% or less. However, this goal should be individualized to balance the effective prevention of microvascular complications with the risk of hypoglycemia [27]. In patients with a reduced GFR, intensive glycemic control might increase the risk of hypoglycemia [28,29]. In advanced stages of CKD in T2D, it is necessary to avoid certain hypoglycemic medications or use them in reduced doses, depending on the degree of kidney function impairment.

Lifestyle modifications, including healthy dietary patterns, regular physical exercise, smoking cessation, and, when applicable, body mass reduction, are recommended for patients with CKD and T2D.

Due to the high CV risk, most patients should be prescribed effective lipid-lowering therapy, primarily based on statin use. Among statins, atorvastatin is often preferred due to its potency and the fact that it does not require dose adjustment based on the GFR [30]. All patients with T2D and CKD or at high risk for CKD, regardless of glycemic control, should be treated with sodium–glucose cotransporter 2 inhibitors (SGLT2i) or with glucagon-like peptide 1 receptor agonist (GLP1RA) semaglutide [31]. Sodium–glucose cotransporter 2 inhibitors (SGLT2i) are usually added to other background antihyperglycemic drugs because SGLT2i has a modest hypoglycemic effect in patients with impaired kidney function. In T2D patients with CKD who do not achieve glycemic control despite initial antihyperglycemic therapy (usually metformin) and SGLT2i, GLP1RA can provide glycemic control (and other metabolic benefits) [32]. GLP1RA are antidiabetic medications that, along with SGLT2i, significantly impact cardiovascular and renal outcomes in patients with pre-existing CV or kidney disease [31].

If albuminuria exceeds 30 mg/day despite the use of an ARB (or ACEi) in maximum tolerated doses and an SGLT2i (or if these agents are not tolerated), finerenone, a non-steroidal selective mineralocorticoid receptor antagonist (MRA), is suggested (unless patients are at high risk for hyperkalemia). Finerenone reduces the progression of kidney dysfunction and the risk of CV event risk in CKD patients with T2D.

Expanding the armamentarium of drugs that provide effective nephroprotection in CKD with T2D justifies the current position statement regarding pharmacological nephroprotection by the Polish Society of Nephrology. We believe the widespread implementation of these recommendations into clinical practice would improve the outcomes of patients suffering from CKD and T2D.

## 2. Antihyperglycemic Drugs

### 2.1. Statement 2.1

#### 2.1.1. Statement 2.1.1

We recommend using sodium–glucose co-transporter type 2 inhibitor (SGLT2i) in chronic kidney disease (CKD) patients with type 2 diabetes mellitus (T2D) or semaglutide to prevent or slow down the progression of CKD beyond the antihyperglycemic properties of these drugs [1A]. We recommend using SGLT2i, which has proven efficacy in CKD patients with T2D: empagliflozin, dapagliflozin, and canagliflozin [1A].

- *Comment to Statement 2.1.1* 

The use of SGLT2i to stop or slow down the progression of CKD in type 2 diabetes is well documented across the entire spectrum of GFR. The cardiovascular outcome trials (CVOTs) that included a significant proportion of patients with normoalbuminuria and normal/near-normal GFR demonstrated that SGLT2i are also effective in preventing or delaying the onset of de novo CKD in diabetic patients.

SGLT2i in the cardiovascular outcome trials (CVOTs)

The *Empagliflozin Cardiovascular Outcome Event Trial in Type 2 Diabetes Mellitus Patients* (EMPA-REG Outcome) remains the first study to demonstrate this effect. Composite renal end-point was defined as the time to onset of de novo albuminuria or an increase in urinary albumin/creatinine ratio (UACR) to > 300 mg/g, doubling of baseline serum creatinine concentration (S_Cr_) with a concomitant decrease in eGFR < 45 mL/min/1.73 m^2^, the commencement of the renal replacement therapy (dialysis, renal transplantation) or renal death. In the EMPA-REG Outcome trial, the patients with stage 3 CKD were well-represented—with 25% of patients having an eGFR of less than 60 mL/min/1.73 m^2^ (with mean eGFR equaling 48 mL/min/1.73 m^2^). The composite renal outcome and its components occurred significantly less frequently in patients randomized to empagliflozin than in those receiving a placebo (most patients in both groups were treated with ACEi or ARB). The risk of doubling baseline S_Cr_ with a concomitant reduction of eGFR < 45 mL/min/1.73 m^2^ was reduced by an impressive 44%. Empagliflozin resulted in an early reduction of eGFR equaling −4 mL/min/1.73 m^2^, which was then reversed after four weeks; the eGFR curves crossed after 52 weeks of treatment, and from this time point until the end of the trial, the mean eGFR in empagliflozin-treated patients remained permanently higher than in those receiving placebo. Early decrease and long-term stabilization of eGFR have been observed in virtually all CVOT (CKD and heart failure) trials with SGLT2i [33,34].

The *Canagliflozin Cardiovascular Assessment Study* (CANVAS), the next CVOT completed in T2D patients, also included patients with a high CV risk profile. As in the case of EMPAREG-Outcome and another seminal trial, i.e., the *Dapagliflozin Effect on Cardiovascular Events* trial (DECLARE-TIMI 58), eGFR was well preserved, with its mean value of 76.7 ± 20.3 mL/min/1.73 m^2^. In this trial, the renal end-points for patients randomizing to canagliflozin or placebo were also defined as time to the UACR progression vs. baseline, a reduction of eGFR of at least 40% vs. baseline, the need for renal replacement therapy, or renal death. Also, in this trial, the superiority of canagliflozin has been proved in the composite renal outcome risk, even though the difference in ≥40% reduction of eGFR between groups cannot be extracted from the core publication and the supplemental data [35]. However, canagliflozin remains the first SGLT2i, with the trial designed to evaluate its impact on kidney outcomes in an advanced CKD, with the primary end-point defined as renal outcome. The *Canagliflozin and Renal Events in Diabetes with Established Nephropathy Clinical Evaluation* Trial (CREDENCE) recruited type 2 diabetics with CKD (mean eGFR of 56 mL/min/1.73 m^2^ indicated stage 3 CKD and a median UACR of almost 1 g/g suggested severe and ongoing kidney injury). More than half of the participants also had CVD disease. Altogether, the CREDENCE cohort represented a very high renal and CV risk profile. The composite renal and CV outcome was defined as ESRD (a decrease in eGFR to less than 15 mL/min/1.73 m^2^, the need for dialysis or renal transplantation), doubling of the baseline S_cr_, renal death, and CV death. Treatment with canagliflozin allowed for a 30% reduction of composite renal and CV outcomes, and most of the renal components (except for renal death) were reduced by 26–40%. Since the commencement of dialysis or renal transplantation is based on subjective clinical judgment, it is worth emphasizing that objectively defined progression of CKD, i.e., doubling of baseline S_cr_ and a reduction of eGFR to less than 15 mL/min/1.73 m^2^, was reduced by 40% [36].

DECLARE-TIMI 58 was the trial that completed the series of seminal CVOTs in T2D, adding the data on another drug, dapagliflozin. The renal and CV outcome was defined as a secondary endpoint in the DECLARE-TIMI 58, which represented individuals with a high CV risk or those already suffering from CVD. The secondary composite renal outcome definition included the following: the time to the onset of permanent, at least 40% reduction of baseline eGFR with its decrease to less than 60 mL/min/1.73 m^2^, ESRD, and renal or CV death. Renal function was best preserved in the DECLARE-TIMI-58 trial compared to EMPAREG-Outcome, CANVAS, and especially CREDENCE, with a mean of 85.2 mL/min/1.73 m^2^. The composite renal and CV outcome was reduced impressively by 47%; each component was also significantly reduced, except for renal death. Despite high mean baseline eGFR, patients with full CKD spectrum (including patients with stage 3 CKD) participated in the trial. The nephroprotective effect was largely independent of baseline eGFR or UACR, as well as of CV co-morbidities or risk factors [37,38].

The trials mentioned above were included in several meta-analyses. Assessing the aggregated data from SGLT2i trials points to their universal nephroprotective potential, as evidenced by their ability to reduce the rate of eGFR loss and delay the onset of an ESRD (need for dialysis or kidney transplantation). It can be concluded that nephroprotective abilities of empagliflozin, dapagliflozin, and canagliflozin, were demonstrated in a broad spectrum of patients with CKD, including those in stage 3b (eGFR 45–60 mL/min/1.73 m^2^) suffering from T2D, CVD or having a high CV risk. Nephroprotection was independent of baseline GFR or UACR. Individual studies and meta-analyses also demonstrate a significantly lower risk of developing acute kidney injury (AKI) and hyperkalemia (both events being Achilles’ heels of ACEi/ARB treatment, especially in doses escalated to fully exert nephroprotective effect) [39,40,41,42,43,44,45].

Except for CREDENCE, the trials mentioned above were designed as CVOT. However, the CREDENCE investigators also defined CV endpoints. Although this is beyond the main scope of this statement, it is worth noting that the reduction of differently defined CV outcomes (including hospitalization due to heart failure, CV death, or even all-cause death) was largely independent of baseline eGFR and UACR values. Even more importantly, in some trials, the trends toward better cardioprotective effects could be observed with increasing UACR and decreasing eGFR [46].

One important trial did not fulfil the promise of the effectiveness of SGLT2i in CKD, namely the *Effect of Sotagliflozin on Cardiovascular and Renal Events in Patients with Type 2 Diabetes and Moderate Renal Impairment Who Are at Cardiovascular Risk* trial (SCORED). Sotagliflozin, which inhibits SGLT1 and SGLT2, effectively reduced CV events in T2D patients with heart failure. In the *Effect of Sotagliflozin on Cardiovascular Events in Patients with Type 2 Diabetes Post-Worsening Heart Failure* trial (SOLOIST-WHF), the most considerable CV benefit was observed in patients with eGFR < 60 mL/min/1.73 m^2^ (median eGFR in this trial was 50 mL/min/1.73 m^2^, ranging between 39.5 and 64.5 mL/min/1.73 m^2^) [47]. The SCORED trial was very ambitious in terms of nephroprotection. The study aimed to demonstrate renal benefit from using sotagliflozin in T2D patients with very advanced stages of CKD, namely with the mean eGFR of 24.0 ± 4.0 mL/min/1.73 m^2^. The study failed to demonstrate such benefit, but the interpretation of its results is not entirely clear: lack of effectiveness of sotagliflozin may suggest no class effect in nephroprotection or indicate a lack of effectiveness of SGLT2i in such an advanced CKD stage [48].

SGLT2i in the renal outcome trials

Trials that followed the series of CVOT, SCORED, and CREDENCE asked two additional vital questions: first, whether SGLT2i would be effective in nephroprotection in patients with advanced CKD (though not as advanced as in the SCORED Trial) and second, whether the nephroprotective effects of SGLT2i would also be apparent in patients without diabetes. Though the latter issue is beyond the scope of this statement and was already discussed in our previously published document [1], we should discuss the two trials that became fundamental for contemporary guidelines in nephroprotection, i.e., *A Study to Evaluate the Effect of Dapagliflozin on Renal Outcomes and Cardiovascular Mortality in Patients With Chronic Kidney Disease* (DAPA-CKD) and *A Multicentre International Randomized Parallel Group Double-blind Placebo-controlled Clinical Trial of EMPAgliflozin Once Daily to Assess Cardio-renal Outcomes in Patients With Chronic KIDNEY Disease* (EMPA-KIDNEY) in the context of T2D (still, together they included more diabetic than non-diabetic patients and are essential for understanding the role of SGLT2i in the treatment of CKD in diabetes).

The DAPA-CKD trial recruited 4304 patients—67.5% suffered from T2D (but 58.3% were classified as having diabetic kidney disease). The mean baseline eGFR was 43.1 mL/min/1.73 m^2^, with only 11% of eGFR exceeding 60 mL/min/1.73 m^2^ (thus, the trial was conducted in patients with advanced CKD). Most patients (75%) were in CKD stage 3 (eGFR ranging between 30 and 60 mL/min/1.73 m^2^). Mean UACR (965 and 934 mg/g) and percentages of participants with UACR > 1000 mg/g (48.7 and 47.9%, in dapagliflozin and placebo groups, respectively) pointed to the high risk of a further progression of CKD and an increased CV risk. Primary composite end-point (renal and CV) was defined as the time to the first event of permanent reduction of eGFR of at least 50% vs. baseline, ESRD (need for dialysis, renal transplantation, permanent eGFR reduction below 15 mL/min/1.73 m^2^), renal death or CV death. The secondary composite end-point was defined identically but with the exclusion of CV death (i.e., was purely “renal”). Primary composite end-point was reduced by 39% in dapagliflozin-treated patients and was even higher, i.e., by 44%, for secondary composite end-point, i.e., after excluding CV death. In addition, dapagliflozin reduced the risk of hospitalization due to heart failure (by 29%), hospitalization due to heart failure and CV death, and—all-cause death (by an impressive 31%) [49]. The results of the DAPA-CKD trial largely resembled those observed for the CREDENCE trial, except for all-cause death (reduction in this end-point not demonstrated for canagliflozin) [36]. Results obtained in the DAPA-CKD trial were essentially the same for diabetics and non-diabetics. However, the trend towards better outcomes upon treatment with dapagliflozin could be noticed for non-diabetic patients. It should be admitted that separate analyses performed on patients in CKD stage 4 demonstrated no renal benefit from the treatment with dapagliflozin (observation consistent with the results of the SCORED trial) [50].

The second vital trial to evaluate renal outcomes in patients with advanced CKD was EMPA-KIDNEY. This study included 6609 patients, but less than 50% suffered from diabetes (in only 2057 of them, diabetic kidney disease was considered the cause of CKD). The initial patient assessment was very detailed—1862 patients had their underlying kidney disease diagnosed based on kidney biopsy (which makes the EMPA-KIDNEY trial the largest ever that included so many patients with biopsy-based diagnosis of CKD). The definition of primary composite end-point differed from that used in the DAPA-CKD trial only in a few details. Progression of CKD was defined as ESRD (the need for dialysis, renal transplantation, or permanent reduction of eGFR to less than 10 mL/min/1.73 m^2^ (in DAPA-CKD—less than 15 mL/min/1.73 m^2^) and permanent reduction of baseline eGFR by at least 40% (in the DAPA-CKD trial: by at least 50%). Renal death and CV death were also included in the primary composite end-point. Key secondary end-points included CV hospitalization, CV death, all-cause hospitalization, and all-cause death. Mean baseline eGFR of 37.5 ± 14.8 mL/min/1.73 m^2^ makes the EMPA-KIDNEY trial the largest study to date evaluating the nephro- and cardioprotective effects of SGLT2i in advanced CKD. As many as 34.2% of patients randomized to empagliflozin and 34.8% of those receiving placebo had their eGFR below 30 mL/min/1.73 m^2^ (translating into 1131 and 1151 CKD stage 4 patients in each group, respectively). Median UACR equaling 412 mg/g (interquartile range: 94–1190 mg/g) also defines patients as having a high risk of CKD progression [38]. The primary composite end-point has been reduced by 28% in empagliflozin-treated patients vs. placebo (hazard ratio—HR 0.72 [95% coefficient interval—CI 0.64–0.82], *p* < 0.001). Statistically significant risk reduction was found in the following detailed composites: all-cause hospitalization, CKD progression, and the onset of ESRD. In contrast to the DAPA-CKD, in the EMPA-KIDNEY trial, all-cause mortality, risk of hospitalized heart failure, and CV death were not reduced in empagliflozin-treated patients. Although the impact of empagliflozin on patient outcome was judged as comparable in diabetic and non-diabetic patients, HR for composite outcome equaled 0.64 (95% CI 0.54–0.77) for diabetics and 0.82 (95% CI 0.68–0.99) for non-diabetics, suggesting more significant benefit in the first group of patients. The potential difference in response to treatment with empagliflozin should probably be explained by the exclusion/inclusion criteria applied. The detailed analysis of this issue is beyond the scope of our statement. Risk reduction in the EMPA-KIDNEY trial was independent of the baseline eGFR (and was significant in ranges: <30, ≥30 to ˂45 and ≥45 mL/min/1.73 m^2^), whereas significantly depended on baseline UACR (being significant in patients with UACR > 300 mg/g but not in the remaining UACR ranges (i.e., ˂30 and 30–300 mg/g) [51].

SGLT2i in the heart failure trials and meta-analysis

Interestingly, seminal trials aimed to determine the effectiveness of SGLT2i in heart failure with reduced or preserved heart failure (*Study to Evaluate the Effect of Dapagliflozin on the Incidence of Worsening Heart Failure or Cardiovascular Death in Patients With Chronic Heart Failure With Reduced Ejection Fraction*—DAPA-HF, *Dapagliflozin Evaluation to Improve the LIVEs of Patients With PReserved Ejection Fraction Heart Failure*—DELIVER, *EMPagliflozin outcomE tRial in Patients With chrOnic heaRt Failure With Reduced Ejection Fraction*—EMPEROR-REDUCED and The *Empagliflozin Outcome Trial in Patients with Chronic Heart Failure with Preserved Ejection Fraction*—EMPEROR-PRESERVED), a substantial percentage of patients (45–50%) suffered from diabetes and mean eGFR in these trials ranged between 61 and 66 mL/min/1.73 m^2^ (CKD stage 3 and 4 prevalence equaled 40–50% patients). The nephroprotective efficacy has been demonstrated across all heart failure SGLT2i trials, with the most spectacular effect observed in the EMPEROR-REDUCED trial [52,53,54,55,56,57,58]. The EMPEROR-REDUCED trial demonstrated that empagliflozin was nephroprotective and decreased the risk of composite renal outcome defined as the need for chronic dialysis, renal transplant, sustained reduction of ≥40% eGFR, or sustained eGFR <15 mL/min/1.73 m^2^ for patients with eGFR ≥ 30 mL/min/1.73 m^2^ at baseline (<10 mL/min/1.73 m^2^ for patients with eGFR < 30 mL/min/1.73 m^2^). The long-term eGFR reduction rate was 1.73 mL/min/1.73 m^2^ per year slower in patients receiving empagliflozin than those on placebo (the effect was apparent despite the initial “acute” dip of eGFR by 4 mL/min/1.73 m^2^ within the first few weeks following randomization). This study seems to be the best-designed heart failure study concerning its “renal” aspect, with careful assessment including also UACR [52,53]. The remaining heart failure trials were less convincing regarding the influence of SGLT2i on renal outcomes, which has been analyzed as secondary end-points—studied SGLT2i appeared neutral regarding nephroprotection, and no differences were observed between diabetics and non-diabetics. Canagliflozin was not studied in a large outcome trial recruiting heart failure patients.

Meta-analysis performed by the Nuffield Department of Population Health Renal Studies Group and SGLT2 inhibitor Meta-Analysis Cardio-Renal Trialists’ Consortium analyzing the nephroprotective effect of SGLT2i in patients with T2D and established CKD randomized to the CREDENCE, SCORED, DAPA-CKD, and EMPA-KIDNEY trials indicated the clear renal benefit (relative risk—RR 0.60 [95% CI 0.53–0.69]), with only one drug (sotagliflozin) and study (SCORED) demonstrating lack of nephroprotective efficacy [41].

GLP1RA in the CVOT and renal outcome trials

GLP1RA was demonstrated repeatedly and universally to slow down UACR in diabetes patients. Most of the CVOT demonstrated that the reduced risk combined renal end-points observed in these trials was reduced almost exclusively due to the slowing down of UACR and/or increasing the number/percentage of patients who regressed from macro- to micro- or normoalbuminuria and from micro- to normoalbuminuria [59,60,61,62]. There are some post-hoc analyses of CVOT showing the possibility of slowing down the rate of eGFR loss when the data were analyzed for particular eGFR/UACR ranges [63,64,65]. Some randomized but unblinded trials also demonstrated the possible effect of slowing down the slope of eGFR loss [66]. Until recently, the data from pivotal trials showing the clear nephroprotective benefit of GLP1RA through impact on both crucial renal end-points, i.e., eGFR loss/doubling of S_cr_ or the risk of ESRD, were lacking. This landscape has been changed recently following the completion and then publication of the *A Research Study to See How Semaglutide Works Compared to Placebo in People With Type 2 Diabetes and Chronic Kidney Disease* (FLOW) trial result [67]. In this study, 3533 participants with T2D were randomized to placebo or subcutaneous semaglutide added to the standard of care. The key inclusion criteria best characterized the group of patients with a very high risk of CKD progression and largely resembled the respective criteria in such pivotal trials of nephroprotection as CREDENCE. They included patients with eGFR of 50 to 75 mL/min/1.73 m^2^ and UACR ranging between 300 and 5000 mg/g or an eGFR of 25 to less than 50 mL/min/1.73 m^2^ and UACR of 100 to 5000 mg/g. The primary outcome has been defined as major kidney disease events: a composite of the onset of kidney failure (dialysis, transplantation, or an eGFR of <15 mL/min/1.73 m^2^), at least a 50% reduction in the eGFR from baseline, or death from kidney-related or cardiovascular causes. ACEi or ARB were used in as many as 95.3% of patients, 61.4% were using insulin, and 15.6% were receiving SGLT2i. Mean eGFR was 47 ± 15.2 mL/min/1.73 m^2^, with most subjects suffering from CKD stage 3a (45–60 mL/min/1.73 m^2^; 29.9%) or CKD stage 3b (30–45 mL/min/1.73 m^2^; 38.4%). All prespecified outcomes: primary and confirmatory secondary ones were achieved and demonstrated reduction of the above-mentioned primary end-point by 24%, kidney-specific component event (i.e., primary end-point without CV) by 21%, death from CV causes by 29%, first CV event by 18%, and all-cause death by 20% (all differences between subcutaneous semaglutide 1 mg once weekly vs. placebo were highly significant). The difference in eGFR slope was lower in semaglutide-treated patients and resulted in a 1.16 mL/min/1.73 m^2^ annual difference as compared to placebo (interestingly, an “acute dip” of eGFR in the semaglutide group could be observed following treatment commencement, which, however, did not exceed −2 mL/min/1.73 m^2^ and eGFR returned to baseline within a few weeks). All the effects on eGFR were accompanied by a 40% reduction of baseline UACR at week 104 (vs. 12% reduction in patients treated with placebo). As might be expected, semaglutide-treated patients experienced 4.1 kg greater body weight loss at week 104 of the trial as compared to patients receiving placebo. In summary, the FLOW trial demonstrated pivotal, multidimensional protection of the kidneys and CV system resulting from the treatment with semaglutide in patients with advanced CKD and T2D. Semaglutide also turned out to be life-saving since it decreased both CV and all-cause mortality. In terms of kidney, protection seems comparable to or better than such drugs as canagliflozin, empagliflozin, and dapagliflozin (as demonstrated in such trials as CREDENCE, EMPA-KIDNEY, and DAPA-CKD—in the two latter trials—in their “diabetic” cohorts). However, their life-saving efficacy can also be compared to one study performed to date in a similar group of patients, namely DAPA-CKD.

#### 2.1.2. Statement 2.1.2

Empagliflozin should not be started in patients with eGFR < 20 mL/min/1.73 m^2^. Dapagliflozin should not be initiated in patients with eGFR < 25 mL/min/1.73 m^2^. Canagliflozin should not be started in patients with eGFR < 30 mL/min/1.73 m^2^. Semaglutide should not be initiated with the intention of nephroprotection in patients with eGFR < 25 mL/min/1.73 m^2^. All mentioned drugs may be continued in patients with eGFR below respective thresholds until dialysis or renal transplantation, if tolerated, for renal and cardiovascular benefits [2B].

- *Comment to Statement 2.1.2* 

As we mentioned in the previous comment, the data on nephroprotective properties of SGLT2i in T2D patients can be derived from two distinct groups of trials: CVOT recruiting mostly patients with excellent kidney function and CKD trials with eGFR inclusive for participation set at 20 mL/min/1.73 m^2^ in the EMPA-KIDNEY, 25 mL/min/1.73 m^2^ in the DAPA-CKD, and 30 mL/min/1.73 m^2^ in the CREDENCE trial [36,49,51]. Concerning heart failure trials performed in patients with preserved or reduced renal function, patients with and without T2D were included. The following eGFR values were defined as exclusion criteria in heart failure trials: eGFR < 20 mL/min/1.73 m^2^ in EMPEROR-REDUCED and EMPEROR-PRESERVED Trials, eGFR < 30 mL/min/1.73 m^2^ in the DAPA-HF trial, and eGFR < 25 mL/min/1.73 m^2^ in the DELIVER Trial—eGFR criteria corresponding to those defined for DAPA-CKD and EMPA-KIDNEY [54,55,56,57,58].

Summary product characteristics (SPC) for three SGLT2i available in Poland state that although empagliflozin, dapagliflozin, and canagliflozin should not be initiated below respective eGFR thresholds, they can still be continued even in advanced (stage 5) CKD until dialysis or renal transplantation are needed. Three important comments must be made on this issue.

First, the need for renal replacement therapy (dialysis or transplantation) is not objectively defined—several clinical circumstances lead practitioners to decide to start dialysis, waitlist patients for transplantation, or start planning the living donation transplantation.

Second, there are also ongoing studies recruiting patients on dialysis—in this group, cardiac outcomes are defined as the key end-points (DAPA-HD—NCT05179668; EMPA-HD—NCT05687058; The RENAL LIFECYCLE—NCT05374291; SDHF—NCT05141552). Results of a pharmacokinetic study completed by Barreto et al. proved that dapagliflozin is only slightly dialyzable during hemodialysis and does not significantly accumulate in the plasma of hemodialysis patients. In this study, it was found that dapagliflozin was well tolerated in hemodialysis patients [68]. De La Flor et al. published a paper describing the case series of 7 diabetic patients with residual kidney function treated with incremental hemodialysis. In these patients, treatments with SGLT2i (5 with dapagliflozin and 2 with empagliflozin) were started three months after initiation of renal replacement therapy. The use of SGLT2i over 12 months appeared to be safe and effective in preserving residual kidney function, reducing residual proteinuria, and lowering interdialytic weight gain. These encouraging results need to be confirmed in a randomized, placebo-controlled study [69].

Third, there is increasing evidence that SGLT2i can be safely used in T2D patients following renal transplantation. The history of renal transplantation was a universal exclusion criterion in all clinical trials with SGLT2i. However, it is tempting to use SGLT2i in kidney transplant recipients to improve metabolic control of T2D (preexisting or new-onset diabetes after transplantation), for nephroprotection and for the treatment of heart failure (heart failure, especially with preserved ejection function, is highly prevalent in transplant recipients, especially those with the history of long-term dialysis). From our experience, SGLT2i are used in everyday practice in kidney transplant patients regardless of underlying nephropathy that led to ESRD and regardless of having diabetes. Several observational (so-called real-life) studies support our experience from transplant clinics. Several papers of relatively low quality and case series have been published on this issue. The one most widely cited is the paper published by Lim et al., which summarized the experience with SGLT2i in diabetic kidney transplant recipients. This study identified 226 T2D transplant recipients in the group of 2083 patients from 6 Korean transplant centers. The study used the propensity score matching methodology: transplant patients receiving SGLT2i were matched with three who had not been treated with SGLT2i. SGLT2i was prescribed not earlier than 90 days following transplantation. The primary composite outcome included all-cause mortality, death-censored graft failure (DCGF), and doubling of baseline S_cr_. An acute dip in eGFR over 10% was also analyzed following the introduction of SGLT2i treatment. During the mean follow-up of 62.9 ± 42.2 months, the SGLT2i-treated group experienced a significantly lower risk of the primary composite outcome when using the multivariate and propensity score-matched models (adjusted HR, 0.43; 95% CI, 0.24–0.78; *p* = 0.006 and adjusted HR, 0.45; 95% CI, 0.24–0.85; *p* = 0.013, respectively). The risks of DCGF and S_cr_ doubling in the SGLT2i group were significantly decreased (HR 0.30; 95% CI, 09–0.98 and HR 0.45, 95% CI, 0.23–0.88, respectively), and overall eGFR remained stable. Only 15.6% of the SGLT2i users showed an acute eGFR dip during the first month, which recovered thereafter [70]. Other small observational trials in patients after kidney transplantation have demonstrated the short-term (up to 1–3 years) efficacy of SGLT2i in reducing proteinuria, stabilizing eGFR trajectory, and even reducing the risk of acute rejection. In many studies (though not universally), SGLT2is was shown to improve metabolic control of T2D, reduce body weight and serum uric acid concentration, and improve hemoglobin blood concentration and hematocrit. Importantly, there was no safety signal of an increased risk of the urinary tract or genitourinary infection in the SGLT2i-treated patients. It must be emphasized that most experts suggest adding SGLT2i after completing three months following transplantation, i.e., when the doses of immunosuppressive drugs are tapered, and the risk of infection becomes lower [71,72,73,74,75,76]. Moreover, several studies are underway in kidney and other solid organ transplant recipients to explore the cardio-renal and metabolic risks and benefits of SGLT2i in these patient groups.

There is no randomized control trial (RCT) specifically targeting dialysis patients with the use of semaglutide. However, there is a case series study (including three patients) on patients in maintenance incremental hemodialysis with T2D that documented that semaglutide treatment has favorable effects on glycemic control, albuminuria, weight, blood pressure control, and preservation of residual kidney function during a 6-month follow-up [77]. We are not aware of any ongoing RCT on the semaglutide therapy in dialysis patients.

Similarly to SGLT2i, no RCT specifically targeting renal transplant recipients was performed with the use of semaglutide and the other GLP1RA, but these drugs are routinely used in the treatment of diabetes also in this group of patients due to their efficacy and safety in a broad range of eGFR; no significant interactions are also known for this drug group with immunosuppressive agents. This statement holds for both T2D preexisting before transplantation and new-onset diabetes after transplantation (NODAT) [78]. Observational single-center trials suggested both the cardio- and nephroprotective impact of GLP1RA in these patients [79,80]. We are not aware of any ongoing RCT on the nephroprotective efficacy of any GLP1RA agonist in transplant recipients.

#### 2.1.3. Statement 2.1.3

We recommend adding SGLT2i or semaglutide to renin–angiotensin system inhibitor (RASi) (angiotensin II receptor blocker—ARB or angiotensin-converting enzyme inhibitor—ACEi) as the first-line nephroprotective agent whenever possible, especially in patients with proteinuria or urinary albumin/creatinine ratio ≥ 30 mg/g [1A]. In cases of contraindications or intolerance to RASi (ARB or ACEi), using these drugs is not a prerequisite to starting SGLT2i or semaglutide therapy [2B].

- *Comment to Statement 2.1.3* 

The use of ACEi or ARBs in CVOT was very high and equaled 80.7% in the EMPAREG Outcome trial, 79.8% in the CANVAS Program (patients with CVD or multiple risk factors for CVD), 80.5% in those without CVD or risk factors for CVD, and 82.2% of patients in the DECLARE-TIMI 58 Trial (patients with CVD or multiple risk factors for CVD) and 80.6% in those without CVD or risk factors for CVD. Patients were well-matched regarding ACEi/ARB use in the SGLT2 and placebo groups [81]. In the DAPA-CKD trial, 98.4% of patients in the dapagliflozin arm and 97.9% in the placebo arm were taking ACEi or ARB; respective numbers equaled 85.7% and 84.6% in the empagliflozin- and placebo-treated patients participating in the EMPA-KIDNEY trial (in both trials—with no difference between diabetics and non-diabetics). All participants of the CREDENCE trial were also required to take ACEi/ARB at baseline. Hence, it seems evident that current knowledge on the nephroprotective effect of SGLT2i in diabetic kidney disease (both early and advanced) is almost entirely based on a dual treatment (SGLT2i added to ACEi/ARB). ACEi/ARB intolerant patients were eligible for DAPA-CKD and EMPA-KIDNEY trials. In the EMPA-KIDNEY trial, the number of patients not using the ACEi/ARB treatment (473 of those assigned to empagliflozin and 508 randomized to placebo) would likely allow for a separate analysis of outcome (not available at the time of submission of this statement) [51]. The clinical efficacy of dapagliflozin and empagliflozin in patients not receiving ACEi/ARB remains largely unknown, and such data will likely be available from real-world observational trials. On the other hand, there is no good reason to suspect that SGLT2i would not be effective in diabetic CKD patients not taking ACEi/ARB due to contraindications or intolerance. The only trial that demonstrated the pivotal efficacy of GLP1RA semaglutide (given subcutaneously once a week) in nephroprotection in patients with T2D and advanced CKD, namely the FLOW trial, also provides data almost exclusively in patients receiving ACEi/ARB. These agents were used by 95.3% of trial participants [67].

#### 2.1.4. Statement 2.1.4

There is no clear evidence to recommend additional measurements of serum creatinine, sodium, and potassium concentration after commencement of treatment with SGLT2i or semaglutide—monitoring of these parameters should follow standard guidelines [expert opinion].

- *Comment to Statement 2.1.4* 

Four critical issues need to be discussed concerning additional assessment of serum creatinine concentration, eGFR, and serum sodium and potassium concentrations after commencing the treatment with SGLT2i. All are related to drug safety. The issue of such an additional assessment has been raised by an “acute dip” of eGFR observed universally across all SGLT2i trials, regardless of an indication and/or stage of CKD. Such a dip usually did not exceed 4–5 mL/min/1.73 m^2^; on average, during the first few weeks following the commencement of treatment, it was always followed by a gradual rise in eGFR and was not accompanied by an increase in serum potassium. Such a short-term reduction of eGFR is clinically not relevant [82]. Moreover, secondary analysis of the DAPA-CKD study documented that long-term nephroprotection of SGLT2i was even more apparent in patients with a more pronounced “acute dip” of eGFR. In this study, those with an acute reduction in eGFR > 10% experienced a long-term eGFR decline of −1.58 mL/min per 1.73 m^2^ per year compared with −2.44 and −2.48 mL/min per 1.73 m^2^ per year among those experiencing a less pronounced early reduction or an increase in eGFR, respectively (*p*-interaction = 0.05) [83].

The Nuffield Department of Population Health Renal Studies Group and SGLT2 inhibitor Meta-Analysis Cardio-Renal Trialists’ Consortium meta-analysis confirmed the phenomenon known from the particular SGLT2i trials and previous meta-analyses. Regardless of the indication, baseline eGFR value or presence/absence of T2D SGLT2i reduced the risk of acute kidney injury (AKI) or remains neutral in this issue. Considering CVOT as well as trials in diabetic and non-diabetic CKD, SGLT2i reduced the risk of AKI (RR 0.63; 95% CI: 0.42–0.97) [41]. A meta-analysis of 24 studies in diabetic patients performed by Leibensperger et al. showed no significant effect of SGLT2i on serum sodium concentration (weight mean difference—WMD = 0.00; 95% CI: –0.03 to 0.33) [84]. As mentioned above (in the comment to Statement 2.1.1), SGLT2i reduces the risk of hyperkalemia. Luo et al., in a network meta-analysis of 27 studies involving 43,589 participants with CKD and diabetes, documented that when SGLT2i was given together with ACEi or ARB, the risk of hyperkalemia was significantly lower than that with ACEi or ARB alone (odds ratio—OR, 0.39; 95% CI, 0.21 to 0.73). Moreover, adding SGLT2i to the combination of MRA and ACEi or ARB reduced the occurrence of hyperkalemia (OR, 0.31; 95% CI, 0.16 to 0.62) [85]. No increased risk of hyperkalemia was observed, particularly in patients with CKD and those with heart failure with reduced ejection fraction on concomitant treatment with MRA [45]. On the other hand, in the above-quoted meta-analysis by Neuen et al., it was found that SGLT2 inhibitors did not increase the risk of hypokalemia [46]. Since in the FLOW trial, AKI episodes occur numerically less frequently, and episodes of hyperkalemia were not reported in the core publication of the trial (though both are unlikely in the treatment with GLP1RA), it seems that the rule of routine renal function and electrolyte monitoring upon treatment with SGLT2i also applies to GLP1RA used in advanced CKD with T2D [67].

### 2.2. Statement 2.2

The target glycated hemoglobin blood concentration (HbA1c) < 7% is recommended to prevent the progression of CKD in patients with T2D. In subjects with a high risk of iatrogenic hypoglycemia, HbA1c of 7–8% is acceptable [2B].

- *Comment to Statement 2.2* 

The target blood HbA1c concentrations that are associated with the best outcome in CKD patients have yet to be well established, and data are mostly interpolated from studies of patients without CKD [86]. All clinical practice guidelines recommend an individualized HbA1c target ranging from 6.5 to 8%, depending on patient-related factors, such as the risk of hypoglycemia, comorbidities, cardiovascular disease, and life expectancy [27,32].

For example: 1. In younger (<50 years) patients with CKD stages 1–2 without significant comorbid conditions, the blood HbA1c concentration goal should range between 6.5 and 6.9%, 2. For patients with CKD stages 3–4, multiple comorbid conditions, and without insulin treatment, the blood HbA1c concentration goal should aim at 7.0–7.5%, 3. For patients with CKD 3–5 on dialysis and treated with insulin, the blood HbA1c concentration goal should be closer to 8%.

Target blood HbA1c concentrations mentioned above were supported by a large Canadian population-based study of 23,296 CKD (eGFR 15–59.9 mL/min/1.73 m^2^) patients with diabetes [87]. Over the median 46-month follow-up, 3665 CKD patients died, and 401 developed ESRD. Regardless of the baseline eGFR, a higher HbA1c blood concentration was strongly and independently associated with an excess risk of all five outcomes studied (death, CKD progression based on a doubling of S_cr_, or new onset ESRD, CV events, all-cause hospitalization) (*p* < 0.001 for all comparisons). However, the association with mortality was U-shaped, with increases in risk apparent at HbA1c blood concentrations lower than 6.5% and higher than 8%. The increased risk of ESRD associated with a higher HbA1c concentration was attenuated at a lower baseline eGFR (*p*-value for interaction < 0.001). Specifically, among those with an eGFR of 30.0–59.9 mL/min/1.73 m^2^, the risk of ESRD was increased by 22% and 152% in patients with blood HbA1c concentrations of 7–9% and higher than 9%, respectively, compared with patients with blood HbA1c concentrations lower than 7% (*p* < 0.001). In contrast, corresponding increases were 3% and 13%, respectively, in those with an eGFR of 15.0–29.9 mL/min/1.73 m^2^. Notably, the excess risk of kidney failure associated with a higher blood HbA1c concentration was most pronounced among people with better kidney function. These findings suggest that appropriate and timely control of blood HbA1c concentration in patients with T2D and CKD may be more crucial than previously comprehended. However, it also suggests intensive glycemic control (HbA1c blood concentration < 6.5%) may be associated with increased mortality.

Two meta-analyses demonstrated that intensive glucose control (target HbA1c 6.1–7.1%) can lead to reduced incidence of albuminuria in people with T2D. However, there was no significant impact on other renal outcomes, such as a doubling of S_cr_, progression to ESRD, or death from kidney disease [88,89].

Another meta-analysis implied that intensive glycemic control had benefits in reducing some renal outcomes. Still, the heterogeneity of glycemic targets limits the validity of that conclusion [90]. In summary, all current recommendations point to the need for individualized pragmatic glycemic goals that balance the benefits and risks of intensive glucose lowering in CKD people with T2D, together with education on hypoglycemia avoidance and self-management.

HbA1c measurement in CKD

Glycated hemoglobin measurement and its interpretation in CKD patients need some comments. Long-term glycemic control in CKD patients with diabetes is assessed with blood HbA1c concentrations as in patients with diabetes and normal kidney function. The linear relationship between the average concentration of serum glucose and blood HbA1c is similar in patients with and without CKD. However, the relationship is weaker in CKD patients with an eGFR < 30 mL/min/1.73 m^2^ [91]. The most important reasons for this inaccuracy in advanced CKD are as follows: 1. Altered red blood cell turnover, especially in patients treated with erythropoiesis-stimulating agents (ESAs); shorter erythrocyte lifespan leads to a greater proportion of younger cells, thus falsely lowering HbA1c values; 2. Analytical (in case of older HbA1c assays) interference of reagent with carbamylated hemoglobin (formed in the presence of an elevated concentration of urea), leading to false elevation in the HbA1c values; laboratories should only use HbA1c assay methods certified by the NGSP (The National Glycohemoglobin Standardization Program).

For these reasons, it is reasonable to use (except for HbA1c level) glucose monitoring either with a glucometer or continuous glucose monitoring systems.

We do not recommend the measurement of glycated albumin for glycemic control in the CKD population [92]. Glycated albumin reflects glycemic control over a much shorter interval (7 to 14 days, compared with 60 to 120 days for HbA1c) and may not be reliable in patients with proteinuria. In addition, it was demonstrated in patients with T2D and CKD undergoing intravenous iron or ESA therapy that HbA1c, compared with glycated albumin and other markers of glycemic control, was most closely associated with mean blood glucose [93]. Only a few long-term clinical trials evaluated the relationship between glycated albumin and the risk of chronic complications of diabetes [94].

### 2.3. Statement 2.3

Antihyperglycemic drugs with proven nephroprotective efficacy or lowering albuminuria properties should be preferred for metabolic control of diabetes. They include SGLT2i (empagliflozin, dapagliflozin, or canagliflozin) [1A] and glucagon-like receptor agonists type 1 (GLP1RA): semaglutide [1A], liraglutide, dulaglutide, lixisenatide, and exenatide [2B]. They may be initiated following metformin, together with metformin, and may be used without metformin. Two- or three-drug regimens (metformin, SGLT2i, and/or GLP1RA) may be applied as an initial treatment or at any time during T2D with CKD [expert opinion].

- *Comment to Statement 2.3* 

As we discussed in comments to Statement 2.1.1, SGLT2i became a cornerstone of nephroprotection in diabetic kidney disease of any stage.

GLP1RAs remain an essential therapeutic option in the broad eGFR range. They seem to be drugs of choice in metabolic control of diabetes and possibly nephroprotection in patients with T2D and advanced CKD who still do not need insulin (only a handful of antihyperglycemic agents may be used in CKD stages 4 and 5, including insulin). GLP1RAs remain the most potent non-insulin agents with antihyperglycemic potential, and two of them (namely semaglutide and dulaglutide) are not contraindicated even in CKD stage 4 [95]. However, the FLOW study, as the first renal outcome trial with 1 mg *sc* of semaglutide, proved its superior nephroprotective (reduction of the kidney-specific component event by 21%) and survival (reduction of all-cause death by 20% and death from CV causes by 29%) benefits in CKD patients with T2D (see chapter “GLP1RA in the CVOT and renal outcome trials”) [67].

GLP1RAs in CVOTs

Most clinical data on the impact of GLP1RA on renal protection in T2D are derived from the CVOT, which included patients with generally well-preserved renal function (no CKD or early stages of CKD on study entry). In some of these trials, the “renal” exclusion criteria were set on eGFR below 60 or even below 30 mL/min/1.73 m^2^, but CKD stage ≥ 3 patients were generally underrepresented in CVOT. Mean or median values of eGFR at the randomization in such seminal trials as *Evaluation of Cardiovascular Outcomes in Patients With Type 2 Diabetes After Acute Coronary Syndrome During Treatment With Lixisenatide* (ELIXA), *Liraglutide Effect and Action in Diabetes: Evaluation of Cardiovascular Outcome Results* (LEADER), *Trial to Evaluate Cardiovascular and Other Long-term Outcomes With Semaglutide in Subjects With Type 2 Diabetes* (SUSTAIN-6), *Exenatide Study of Cardiovascular Event Lowering Trial* (EXSCEL), *Effect of Albiglutide, When Added to Standard Blood Glucose Lowering Therapies, on Major Cardiovascular Events in Subjects With Type 2 Diabetes Mellitus* (HARMONY) and *Researching Cardiovascular Events With a Weekly Incretin in Diabetes* (REWIND) (CVOT analyzing lixisenatide, liraglutide, semaglutide, exenatide, albiglutide and dulaglutide) ranged between 75 and 80 mL/min/1.73 m^2^ [96,97]. For this reason, patients included in these trials did not represent the high-risk profile for CKD progression defined as doubling of baseline S_cr_, eGFR decreased by ≥40 or 50%, or especially—progression to ESRD.

The renal outcomes in patients receiving liraglutide vs. placebo in the LEADER trial have been published separately in the *New England Journal of Medicine* [59]. In this trial, 9340 patients with T2D and a high CV risk have been randomized in a 1:1 ratio to daily liraglutide injected subcutaneously or placebo (added to the standard-of-care). The composite renal outcome has been defined as time to develop macroalbuminuria de novo, doubling baseline S_cr_ with a permanent decrease in eGFR ≤ 45 mL/min/1.73 m^2^, need for renal replacement or renal death. Liraglutide reduced composite renal outcome by 22% vs. placebo, and the difference was even more pronounced in patients with baseline eGFR exceeding 60 mL/min/1.73 m^2^ (32% reduction). It must be emphasized that this effect has been achieved entirely owing to the decrease in the onset of de novo albuminuria. The rate of eGFR loss was slower in liraglutide-treated patients, especially those with eGFR ranging between 30 and 59 mL/min/1.73 m^2^ (eGFR loss with the rate of −2 mL/min/1.73 m^2^ during 36-month follow-up compared to −4 mL/min/1.73 m^2^ in those receiving placebo). It must be acknowledged that such a rate of eGFR loss is extremely low even in a placebo group and much slower than typically observed in CKD T2D patients [98]. An unexpectedly low rate of the UACR increase in the study (1.8 mg/g in liraglutide-treated patients and 6.3 mg/g in those receiving placebo over 36 months of follow-up) strengthens the impression of an extremely low risk of CKD progression in this trial (though even such a slow rate of UACR increase was further significantly attenuated by using liraglutide). The baseline mean eGFR was 80 mL/min/1.73 m^2^, and only 20.7% of patients with eGFR range between 30 and 59 mL/min/1.73 m^2^ (with just 2.4% with eGFR < 30 mL/min/1.73 m^2^) and micro- and macroalbuminuria present in 26.3 and 10.5% patients, respectively, make this seminal CVOT not representative for the “real life” CKD cohort [59]. Nevertheless, since CV protection was independent of eGFR (and was most pronounced in subjects with eGFR ranging between 30 and 60 mL/min/1.73 m^2^), liraglutide could be considered a life-saving drug also for T2D patients with CKD [59,60].

The SUSTAIN-6 trial investigators defined renal composite outcome using the same components as in the LEADER trial, this time in the study assessing the effectiveness of injectable semaglutide vs. placebo in nephroprotection. The CKD stage 3 or higher was one of the inclusion criteria defining high CV risk in this trial. Composite renal outcome occurred in 3.8% of patients receiving semaglutide and 6.1% of those randomized to placebo (highly significant, 36% risk reduction). This effect was achieved entirely owing to the reduction of a new onset macroalbuminuria (HR 0.54 [95% CI 0.37–0.77]; the risks of developing all remaining components of the composite renal outcome were identical in both groups. Since 71.5% of patients were characterized with eGFR > 60 mL/min/1.73 m^2^, SUSTAIN-6 patients also displayed rather low risk of CKD progression [62].

The LEADER and SUSTAIN-6 investigators proposed the joint analysis of both trials concerning their nephroprotective potential. Both analyzed injectable GLP1RAs reduced the risk of UACR increase by 24% within a 2-year follow-up (with the highest efficacy of semaglutide 1 mg injected once weekly). Semaglutide 1 mg and liraglutide reduced the eGFR loss rate by 0.78 and 0.26 mL/min/1.73 m^2^ per year, respectively, vs. placebo (both significant differences) [63].

Similar results were obtained when studies with two forms of semaglutide, i.e., once-weekly subcutaneous and daily oral, were analyzed (pooled analysis of renal outcomes for the SUSTAIN-6 and PIONEER-6 trials). Both studies involved 6480 patients randomized to treatment with semaglutide or placebo. Patients had well-preserved renal function with the incipient diabetic kidney injury, as reflected by a mean eGFR of 75 ± 21.8 mL/min/1.73 m^2^ and a median UACR of 24.7 mg/g. Treatment with one of the semaglutide formulations, even in such an early stage of CKD, slowed the progression of CKD (eGFR reduction –0.97 mL/min/1.73 m^2^ per year compared to −1.56 mL/min/1.73 m^2^ in placebo-treated patients, translating into a statistically and clinically significant difference equaling 0.59 mL/min/1.73 m^2^ per year). Of note, such a reduction in an eGFR decrease rate was most apparent in patients with baseline eGFR ranging between 30 and 60 mL/min/1.73 m^2^, i.e., those at the highest risk of progression (the between-group difference was 1.06 mL/min/1.73 m^2^ per year). Both semaglutide formulas significantly reduced albuminuria [64].

The REWIND trial further expanded our knowledge of the role of GLP1RA in nephroprotection. In this study, patients with T2D were randomized to dulaglutide 1.5 mg weekly or placebo added to the standard of care. All included patients had high CV risk or a history of CV; eGFR as low as 15 mL/min/1.73 m^2^ was an exclusion criterion (but—despite setting such a liberal range of eGFR—only in 1% of eGFR was lower than 30 mL/min/1.73 m^2^, in 22.2%—lower than 60 mL/min/1.73 m^2^, and mean eGFR was 76.6 ± 22.8 [placebo] and 77.2 ± 22.7 mL/min/1.73 m^2^ [dulaglutide]). UACR of 1.80 and 1.88 mg/mmol, respectively (translating into 15.9 and 16.6 mg/g), i.e., well below microalbuminuria cut-off value together with well-preserved renal function point on the incipient stage of diabetic kidney injury characterizing the REWIND trial cohort. Standardized (only slightly modified as compared to other trials) composite renal end-point included the onset of macroalbuminuria > 33.9 mg/mmol (>300 mg/g), reduction of baseline eGFR by at least 30%, or ESRD (need for dialysis or renal transplantation). The risk of this end-point decreased by a statistically significant 15% (the study included 9901 patients with a median follow-up of 5.4 years). As might be expected, the therapeutic effect of dulaglutide mostly relied on preventing the increase in UACR. The composite renal end-point has been reduced regardless of an initial eGFR (was significant in subjects with eGFR below and above 60 mL/min/1.73 m^2^), regardless of baseline UACR (normo-, micro-, macroalbuminuria) and regardless of the concomitant treatment with ACEi/ARB. However, the independent nephroprotective effect of dulaglutide was attenuated after correcting for differences in blood pressure (BP) values and blood HbA1c concentration observed between the groups. Dulaglutide was demonstrated to diminish the risk of eGFR decrease by 40% (HR 0.70 [95% CI 0.57–0.85]) and by 50% (HR 0.56 [95% CI 0.41–0.76]) vs. baseline [64].

Another study with dulaglutide was the AWARD-7 trial. The study group reproduced the real cohorts of T2D patients attending the nephrology outpatient clinics. Depending on study subgroups, 93–96% were characterized with eGFR < 60 mL/min/1.73 m^2^, and in 30%, eGFR was in the range of 15 and 30 mL/min/1.73 m^2^ (mean for the whole study group—36 mL/min/1.73 m^2^), adding the median UACR between 195.6 and 233.6 mg/g points on the advanced CKD with a high risk for further progression. Patients were randomized to receive dulaglutide 1.5 mg weekly, dulaglutide 0.75 mg weekly, or insulin glargine. The efficacy of dulaglutide vs. insulin in improving the metabolic control of diabetes comprised the primary end-point, but renal outcome has also been carefully evaluated. As compared to CVOTs, the AWARD-7 trial was much smaller in terms of patient number included (dulaglutide 1.5 mg—192 patients; dulaglutide 0.75 mg—190 patients; insulin glargine—194 patients), and the observation period was just 52 weeks. Interestingly, two eGFR formulas were used to assess renal function: CKD-EPI based on serum creatinine (recommended in most modern guidelines) and CKD-EPI based on serum cystatin C (considered more precise by some authors). The study was randomized, but treatment with insulin or dulaglutide was unblinded (both cohorts using dulaglutide were blinded against the baseline dose). The eGFR remained stable in patients receiving both doses of dulaglutide in weeks 26 and 52, whereas it started to decrease in the insulin glargine cohort. At the study completion, eGFR was lower by 2.9 mL/min/1.73 m^2^ vs. baseline in the insulin glargine group, whereas respective decreases in patients receiving the lower or higher doses of dulaglutide were 1.1 and 1.5 mL/min/1.73 m^2^, respectively (differences between the insulin and GLP1RA groups were even more profound when the cystatin C-based formula was applied). eGFR reduction was even faster in subjects with macroalbuminuria receiving insulin (−5 mL/min/1,73m^2^ in week 52). UACR reduction was very fast in both dulaglutide-treated cohorts and was proportional to the baseline UACR. For example, after 26 weeks of treatment, UACR was reduced by 43.1% in subjects receiving dulaglutide 1.5 mg and 25.3% in those receiving 0.75 mg if baseline UACR exceeded 300 mg/g. UACR remained stable in the insulin-glargine-treated patients [66].

“Twincretin” agent (tirzepatide) in the renal outcome trial

Tirzepatide is the first therapeutic agent that stimulates two receptors engaged in the incretin action, namely GLP1 and GIP (“twincretin”). In the series of trials with the common acronym SUPRASS, several benefits of tirzepatide in terms of metabolic control of diabetes and weight loss were demonstrated—the drug appeared to be more effective as compared to placebo, insulin degludec and glargine, and even as compared to semaglutide and dulaglutide [64,99,100,101,102,103]. The CVOT trial for tirzepatide was not yet published or completed. The most attractive for the nephrologist is the SURPASS-4 trial, an open-label, randomized phase III trial in which three doses of tirzepatide (5, 10, and 15 mg given once weekly sc) were compared with insulin glargine. The primary end-points were focused on the metabolic control of diabetes, but several renal end-points were also analyzed in the trial. Nine hundred ninety-seven patients were randomized to the tirzepatide group (one of the three doses), and 1005 received insulin. The mean follow-up was 84 weeks, with a maximum of 104 weeks. Considering the metabolic efficacy of tirzepatide, it appeared better than insulin in diabetes control. The trial recruited patients with a high CV risk, and low eGFR (eGFR < 60 mL/min/1.73 m^2^) was considered as such a risk factor (though the mean eGFR in the study cohort equaled 81.3 ± 21.1 mL/min/1.73 m^2^ and only 17% of patients suffered from CKD stages 3 or 4). Eight percent of patients were characterized with overt albuminuria, and in 28%, a UACR ranged between 30 and 300 mg/g (median value of 15 mg/g). In addition, the investigators defined the group of patients with a high risk of CKD progression as eGFR < 75 mL/min/1.73 m^2^ with UACR >300 mg or eGFR < 45 mL/min/1.73 m^2^ regardless of UACR. The composite renal outcome was defined as the time to reduce eGFR by at least 40%, ESRD, macroalbuminuria de novo, and renal death. More than 80% of patients were treated with ACEi or ARB and 25% with SGLT2i. The slope of an eGFR in this trial was similar to that observed in SGLT2i-treated patients. The ‘acute dip’ (mean decrease of –2.1 mL/min/1.73 m^2^) in the first few weeks and then its gradual reverse (from week 12 until the end of a trial, the eGFR values in tirzepatide-treated patients were higher as compared to those receiving insulin). The eGFR loss rates equaled—1.4 ± 0.2 mL/min/1.73 m^2^ per year in tirzepatide-treated patients vs.−3.6 ± 0.2 mL/min/1.73 m^2^ per year in those receiving insulin (the difference is highly statistically significant). The impact on eGFR was independent of using ACEi/ARB/SGLT2 and from the metabolic control or the weight loss rate. Even more importantly, all renal benefits were apparent and even more pronounced in patients with a baseline eGFR < 60 mL/min/1.73 m^2^. Concerning UACR, treatment with tirzepatide allowed for UACR reduction by 6.4% in week 52 and was 4.4% lower vs. baseline at the study end. An increase in UACR by 24.1% in week 52 and 56.7% in week 104 vs. baseline was observed in the insulin-treated patients (the difference in UACR is highly significant between the two treatment groups). The composite renal end-point has been reduced by an impressive 42% (HR 0.58 [95% CI 0.43–0.80]), mostly owing to the lower risk of UACR increase. As for today, the knowledge of the nephroprotective effectiveness of tirzepatide is incomplete, but we should expect further trials, hopefully confirming and expanding the findings of a SURPASS-4 study [103].

Aspects of co-antihyperglycemic treatment and metabolic control in CVOTs with SGLT2i and GLP1RA

In CVOT evaluating the usefulness of SGLT2i and GLP1RA, the respective agents or placebo were added to other antihyperglycemic drugs (the standard of care). In most of the trials, patients suffering from T2D included in CVOT, heart failure or CKD trials with SGLT2i and CVOT with GLP1RA might be naive to antihyperglycemic treatment (this was not an infrequent scenario in some trials—for example, in the EMPAREG—Outcome Trial only 19.5% of patients in the empagliflozin arm and 31.5% of patients in the placebo arm were taking any glucose-lowering therapy) or could be treated with one or more antihyperglycemic drugs (for example, in the LEADER trial treated patients 88.1% in liraglutide and 88.4% placebo arms with 21.7 and 29.1% of patients with respective arms having the new antihyperglycemic agent introduced during trial, and in the SUSTAIN-6 trial 99% of all study subjects received such treatment at baseline). It is worth emphasizing that in CVOT with SGLT2i and GLP1RA, the baseline metabolic control of diabetes was defined as an inclusion criterion. In contrast, this issue was considered far less important in the heart failure and CKD trials performed with SGLT2i. A baseline HbA1c range was not defined as an inclusion or exclusion criterion in both CKD trials. Type 1 diabetes and a history of ketoacidosis were the only diabetes-related exclusion criteria. To the best of our knowledge, in core publications of both trials the data on concomitant anti-hyperglycemic agents in T2D patients, as well as the renal outcome depending on the type and number of such drugs received by patients, were not reported.

The overview of the critical inclusion criteria and antihyperglycemic treatment in the trials discussed in this comment is summarized in Table 1. As can be concluded from Table 1, the study populations significantly differed in terms of the percentage of patients receiving antihyperglycemic treatment and the type and number of drugs they were taking. The inclusion criteria concerning the baseline metabolic control also significantly differed and might be quite restrictive (e.g., REWIND trial) or very broad and liberal (e.g., DECLARE-TIMI 58 and CREDENCE trials). In general, patients in the GLP1RA trials were more heavily pretreated than those in the SGLT2i trials. The types of drugs used in respective studies also mirror the progress in T2D management. In more recent published studies, the percentage of patients receiving sulfonylurea agents decreased, whereas those treated with DPP4i and SGLT2i (in GLP1RA studies) or GLP1RA (in SGLT2i studies) increased. Data on T2D treatment and metabolic control must be considered when interpreting the particular study’s findings. There is no doubt that SGLT2i and GLP1RA offer a synergistic effect in metabolic control when coupled with other antihyperglycemic agents. Whether such a synergy is also true for nephroprotection remains an open item. We have found one study comparing the effectiveness of empagliflozin added to the other non-SGLT2i antihyperglycemics vs. non-SGLT2i antihyperglycemics on the risk of major adverse kidney events (MAKE) of eGFR decline >50%, end-stage kidney disease, or all-cause mortality among 379,033 people with T2D and a full spectrum of CKD (mean eGFR 77.5 mL/min/1.73 m^2^) [104]. Empagliflozin use was associated with 0.99 mL/min/1.73 m^2^ (95% CI 0.51, 1.55) less annual reduction in eGFR, 0.25 kg/m^2^ (95% CI 0.16, 0.33) more annual decrease in BMI, and reduced risk of MAKE (hazard ratio [HR] 0.68 [95% CI 0.64, 0.73]).

We conclude that available data point to the effectiveness of SGLT2i and GLP1RA in nephroprotection regardless of concomitant treatment for T2D—the agents from both groups can be added to already existing therapy and may be used as the first antihyperglycemic medications (Table 1). The therapy combining SGLT2i, GLP1RA and one or more other agents (oral and injectable) can also be used for achieving metabolic control, CV protection and nephroprotection, depending on an individual patient’s risk profile.

### 2.4. Statement 2.4

Antihyperglycemic drugs needed to achieve target HbA1c in CKD patients with T2D should be chosen based on eGFR with dose adjustment and treatment cessation when necessary according to eGFR. Patients should be carefully monitored to detect possible side effects of these drugs (with particular attention paid to hypoglycemia) [2B].

- *Comment to Statement 2.4*

Pharmacological therapies for treating diabetes include oral agents, non-insulin injectable agents and insulin. The choice of agent depends upon glycemic goals, the stage of CKD, the risk of medication-associated adverse events (lactic acidosis and hypoglycemia—the risk of both increases as eGFR declines), patient comorbidities, preference, and convenience [29,105,106,107]. In addition, given the risk of renal function deterioration over time, regular monitoring of eGFR is necessary, as this could impact the type and dosage of antihyperglycemic therapies and the appropriate glycemic target. Simultaneously, it should be noted that calculated eGFR (we advocate a CKD-EPI formula rather than MDRD) may not reflect an actual kidney function, e.g., in obese or malnourished patients, those who underwent amputations, etc. In such cases, calculating eGFR using serum cystatin C-based formulas or creatinine clearance using the Cockcroft–Gault formula should be preferred [27,107]. Appropriate combinations of different medication classes will frequently be needed to manage CKD patients with T2D, and the choice of drugs needs judicious consideration (Table 2).

Metformin

- Metformin can be used if an eGFR is not lower than 30 mL/min/1.73 m^2^. The dosage should be reduced when the eGFR falls below 45 mL/min/1.73 m^2^ (to a maximum of 1000 mg per day) [108].
- There are two reasons to avoid metformin in patients with eGFR < 30 mL/min/1.73 m^2^:
  ○ an increased risk of lactic acidosis was demonstrated in some studies [109], although not universally confirmed [110],
  ○ lack of high-quality evidence for the benefits of continuing metformin in patients with eGFR lower than 30 mL/min/1.73 m^2^. Many studies demonstrated that metformin use in patients with T2D and eGFR > 30 mL/min/1.73 m^2^ confers a survival benefit (22% reduction of mortality; HR 0.78; 95% CI 0.63–0.96) and a 30%–40% reduction in cardiovascular and diabetes-related events [111].
- Metformin should be withheld during acute illnesses with a risk of tissue hypoxia or sudden deterioration in renal function, particularly in AKI and sepsis [112].
- It is not necessary to discontinue metformin before intravenous contrast media administration [2].
- During metformin therapy, attention should be paid to vitamin B_12_ deficiency [29].

Sulfonylureas

- Patients with T2D and CKD < 60 mL/min/1.73 m^2^ who are on sulfonylureas treatment (with or without concomitant insulin therapy) are at increased risk of hypoglycemia.
- Sulfonylureas with the lowest risk of hypoglycemia and hepatic metabolism (glipizide, glimepiride, gliquidone, and gliclazide) are reasonable agents for patients with an eGFR > 30 mL/min/1.73 m^2^.
- Most sulfonylureas should be avoided in advanced renal impairment (eGFR < 30 mL/min/1.73 m^2^). The only sulfonylurea agent that might be safely used in patients with eGFR < 30 mL/min/1.73 m^2^ is metabolized mainly in the liver to largely inactive metabolites, gliquidone.
- Given the lack of excess cardiovascular events and a lower risk of doubling S_cr_ in patients with eGFR ≥ 60 mL/min/1.73 m^2^ (HR: 0.21, 95% CI: 0.04–0.99), gliclazide should be a drug of choice in these patients, i.e., patients with normal kidney function [113].

Meglitinides

- Repaglinide, due to its hepatic metabolism (via cytochrome P450), can be considered for T2D therapy in CKD patients as monotherapy or in addition to metformin.
- Nateglinide is hepatically metabolized with renal excretion of active metabolites that can accumulate and cause hypoglycemia. Therefore, it should be avoided in patients with advanced CKD or ESRD.
- Repaglinide dose reduction is advised in patients with eGFR < 30 mL/min/1.73 m^2^ [29].

Thiazolidinediones: Pioglitazone

- Patients with T2D and CKD of all stages can be considered for treatment with pioglitazone.
- Pioglitazone should be avoided in patients with advanced CKD and fluid overload, especially those with preexisting heart failure, given the risk of edema and worsening heart failure [29].
- We suggest discontinuing pioglitazone in CKD patients with T2D, experiencing hip fracture during treatment, or with painless hematuria until bladder cancer is excluded [29].

Dipeptidyl Peptidase-4 (DPP4) Inhibitors

- Patients with T2D and CKD of all stages, including those requiring dialysis, can be considered for treatment with DPP4 inhibitors without the risk of hypoglycemia [114].
- DPP4 inhibitors appear to have a neutral effect on the risk of diabetes-related kidney disease and kidney outcomes [29].
- Doses of DPP4 inhibitors (sitagliptin, saxagliptin, and vildagliptin) should be appropriately reduced by the degree of renal impairment [29].
- We recommend linagliptin, which is only minimally excreted in the urine (less than 10 percent), as a drug of first choice due to the lack of need to adjust its dose to eGFR [115,116].

Sodium–Glucose Co-Transporter-2 Inhibitors (SGLT-2 Inhibitors)

See Statement 2.1.1., 2.1.2 and 2.1.3

Glucagon-like Peptide-1 Receptor Agonists (GLP-1RAs)

See Statement 2.1.1., 2.1.2 and 2.1.3.

Alpha-Glucosidase Inhibitors A

- Acarbose, or miglitol, can be safely used in patients with all CKD stages [117].
- They are minimally effective in lowering blood HbA1c concentration (mean 0.5 to 0.7% reduction) and are associated with limiting gastrointestinal side effects [117].

Insulin

The indications for initiating insulin therapy and the principles underlying insulin therapy are the same in patients with non-dialysis CKD as in the general diabetic population. However, insulin doses must be higher in early CKD stages, when insulin resistance predominates. As eGFR declines, however, insulin requirements diminish. Some studies suggest a 30% reduction in insulin requirements when the eGFR is <60 mL/min/1.73 m^2^, compared with when the eGFR is >90 mL/min/1.73 m^2^. This phenomenon results from the decreasing renal catabolism of insulin (healthy kidneys clear up to 30% of this hormone through glomerular filtration followed by tubular reabsorption and breakdown). There is no evidence that insulin therapy reduces the risk of progressive renal disease [29,118].

Co-Formulation

GLP-1RAs and basal insulin co-formulations are now available: liraglutide with insulin degludec (Xultophy^TM^) and lixisenatide with insulin glargine U100 (Suliqua^TM^). Their renal limitations are those of their respective GLP-1RAs. There are no specific trials of these combinations in patients with T2D and CKD.

## 3. Antihypertensive Therapy

### 3.1. Statement 3.1

#### 3.1.1. Statement 3.1.1

We suggest that patients with CKD and T2D with hypertension should be treated to the same target office blood pressure (BP) as other patients with hypertension, i.e., to 130–139/70–79 mmHg and perhaps lower (120–129/70–79 mmHg) if tolerated [expert opinion].

- *Comment to Statement 3.1.1* 

Hypertension is a common comorbidity in T2D [119,120]. Many studies have shown that BP control reduces CV morbidity and mortality rates, as well as CKD progression [120,121,122]. Hence, managing high BP is a significant task in CKD patients, with two primary objectives: prevention of CV events and protection against CKD progression. On the other hand, too aggressive antihypertensive therapy, leading to hypotension, might have adverse effects on kidney function [123].

Several studies, including those discussed below, have analyzed the effects of antihypertensive therapy on the development of CKD in T2D patients. However, their results are inconclusive, and the target BP remains an open question.

The *Appropriate Blood Pressure Control in Diabetes* (ABCD) trial included 480 diabetic participants and evaluated the effects of intensive versus moderate BP control on vascular complications. The mean BP value within the four years was 128 ± 0.8/75 ± 0.3 mm Hg for the intensive antihypertensive therapy group and 137 ± 0.7/81 ± 0.3 mm Hg for the moderate antihypertensive therapy group (*p* < 0.0001). All-cause mortality was reduced in the intensive BP control patients compared with the moderate control patients. There was no difference in kidney function between the above-mentioned group of patients. However, a significantly lower percentage of patients progressed to albuminuria (*p* < 0.02) and from albuminuria to severe albuminuria (*p* < 0.02) in the intensive therapy group was found [124].

The *UK Prospective Diabetes Study* 38 (UKPDS 38) showed that tight BP control in T2D patients with hypertension has led to a reduction in the risk of diabetes-related death. The mean BP achieved in this study in patients over nine years of follow-up was 144 ± 14/82 ± 7 mmHg under tight control and 154 ± 16/87 ± 7 mmHg under less tight control (*p* < 0.0001). In the tight control group, the 24% reduction in diabetes-related end-points (95% confidence interval: 8% to 38%; *p* = 0.0046), 32% reduction in diabetes-related deaths (6% to 51%; *p* = 0.019), 44% reduction in stroke (11% to 65%; *p* = 0.013), and 37% of microvascular end-points (11% to 56%; *p* = 0.0092) were observed. There was a non-significant reduction in all-cause mortality. No significant difference in kidney function or the proportion of patients who had doubling baseline S_cr_ between both groups was noticed [125]. It should be mentioned that the S_cr_ > 175 µmol/L was the exclusion criterion from the study.

The *Action to Control Cardiovascular Risk in Diabetes* (ACCORD) trial that enrolled patients with T2D did not show a difference in the composite CV events between the intensive systolic blood pressure (SBP) target (<120 mm Hg) and standard SBP target (<140 mm Hg). However, the intensive BP control demonstrated a significant reduction in the risk of stroke (HR: 0.59; 95% CI: 0.39–0.89) but was associated with a more frequent serious adverse event rate. In the ACCORD study, patients with serum creatinine concentration >1.5 mg/dL (132 µmol/L) were excluded, and in 36%, CKD was defined by albuminuria only. The patients in the intensive SBP group had lower mean eGFR at the final study visit as compared with the standard BP control group (74.8 ± 25.0 vs. 80.6 ± 24.8 mL/min/1.73 m^2^) with a similar incidence of a primary microvascular composite outcome of renal failure and retinopathy (11.4% vs. 10.9%) and ESRD (2.5% vs. 2.4%) [126]. The cumulative incidences of CKD in ACCORD intensive and standard SBP groups were 10.0% and 4.1%, respectively. It was shown that intensive SBP lowering resulted in a higher risk of impaired kidney function [127]. 

On the other hand, the *Irbesartan Diabetic Nephropathy Trial* (IDNT) showed renal benefits from lower achieved BP values. The inclusion criteria for this study came complete with baseline serum creatinine concentration up to 265 μmol/L (3.0 mg/dL) and urine protein excretion > 900 mg per 24 h. Baseline average BP was 159 ± 20 and 87 ± 11 mmHg. During a median follow-up of 2.6 years, SBP > 149 mmHg was associated with a 2.2-fold increase in the risk for doubling serum creatinine or ESRD compared with SBP < 134 mmHg. Renal outcomes in patients with SBP < 120 mmHg were not substantially better than those with SBP between 120 and 130 mmHg, while all-cause mortality increased below SBP < 120 mmHg [128,129]. 

The impact of BP on kidney impairment markers was also assessed in the *Action in Diabetes and Vascular Disease: PreterAx and DiamicroN-MR Controlled Evaluation Study* (ADVANCE). The study showed that the risk of renal events was reduced at a lower BP value (mean SBP 134.7 vs. 140.3 mm Hg). Moreover, the lowest risk for renal events in this study was observed among participants with achieved SBP < 110 mmHg or diastolic blood pressure (DBP) 65 mmHg [130].

However, the observational subgroup analysis of participants in the *International Verapamil SR-Trandolapril Study* (INVEST) showed that tight control of SBP (<130 mmHg) among patients with T2D and coronary artery disease was not associated with improved CV outcomes compared with usual control (SBP > 140 mmHg). All-cause mortality for at least two years was 22.8% in the tight control vs. 21.8% in the usual control group (adjusted HR 1.15; 95% CI, 1.01–1.32; *p* = 0.04) [131].

The meta-analysis of 17 RCTs included only patients with diabetes, and 24 RCTs separately reported data for patients with and without diabetes, assessing the effects of lowering SBP on CV events and ESRD development. A significant kidney benefit could only be detected when the baseline SBP was >140 mmHg (SBP 150–140 mmHg: 44% RR reduction, 45 ESRD cases avoided in 1000 patients treated for five years). No significant ESRD risk reduction was found at lower SBP, particularly <130 mmHg. Additionally, in diabetes, most of the CV risk reduction occurs in patients with DBP values between 80 and 90 mmHg [132].

The meta-analysis of Wang et al. comprising 16 RCTs conducted in hypertensive T2D patients showed no clear benefit of intensive BP lowering compared with less intensive BP lowering (mean achieved 136.6/76.7 mmHg vs. 144.9/81.1 mmHg) on the risk of ESRD (RR, 1.00, 95% CI, 0.75–1.33, *p* = 0.994), but intensive BP lowering reduced the risk of albuminuria progression by 9% (RR, 0.91, 95% CI, 0.84–0.98, *p* = 0.01). Furthermore, in this meta-analysis, the authors showed that intensive BP lowering resulted in a significant reduction in the all-cause mortality risk, major CV events, myocardial infarction (MI), stroke, and CV death [133].

Taking into account all these inconsistent results and the fact that most of the studies did not show unambiguous beneficial effects of a more intensive reduction in BP on the progression of CKD and cardiovascular outcomes, we suggest that T2D patients with CKD and hypertension achieve the same target office BP as in the general population and in patients with non-diabetic CKD, which we included in the recent recommendations of the Polish Society of Nephrology [1].

#### 3.1.2. Statement 3.1.2

We suggest BP measurements in both the recumbent/sitting and standing positions to identify patients with orthostatic hypotension and, consequently, to individualize target office BP in patients with orthostatic hypotension [expert opinion].

- *Comment to Statement 3.1.2* 

Orthostatic hypotension is defined as a decline in SBP of at least 20 mmHg or DBP of at least 10 mmHg within 3 min of standing. It is associated with an increased risk of mortality and micro- and macrovascular complications, CV events, or injurious falls [134,135]. The T2D is a risk factor for orthostatic hypotension [135]. BP in this population should be measured in the sitting and standing positions at the first visit and each visit in older patients treated with antihypertensive drugs. At least two BP measurements should be taken after one and three minutes of standing, particularly when patients are treated with vasodilators [136].

### 3.2. Statement 3.2

#### Statement 3.2.1

Antihypertensive drugs should be started in most CKD patients with T2D and hypertension without unnecessary delay, together with lifestyle modifications [expert opinion].

- *Comment to Statement 3.2.1* 

T2D is associated with a higher rate of resistant hypertension and is recognized to be one of the most critical factors that can make the achievement of BP control difficult [137]. Furthermore, patients with diabetes are considered to be at a high CV risk, which may significantly increase the incidence of established CVD or advanced CKD. Consequently, hypertensive patients with T2D are candidates for immediate initiation of antihypertensive drug treatment together with lifestyle interventions, including a low-salt diet, exercise, smoking cessation, and weight control [138].

### 3.3. Statement 3.3

#### Statement 3.3.1

We recommend using renin–angiotensin system inhibitors (RASi) (angiotensin receptor blockers—ARB or angiotensin-converting enzyme inhibitors—ACEI) as first-line antihypertensive therapy in hypertensive patients with CKD and T2D [1B].

- *Comment to Statement 3.3.1* 

Few randomized clinical trials in patients with T2D pointed to ARB (losartan and irbesartan) as the first treatment choice in hypertensive CKD patients, especially in those with moderate or severe albuminuria, because of their additional well-documented potential to slow down the eGFR loss in T2D (discussed in detail in Statement 4.1.1). Alternatively, other ARB and ACEI might also be used. However, nephroprotective properties of agents other than losartan and irbesartan in T2D need to be better documented.

The previously mentioned IDNT trial, comparing the angiotensin II receptor antagonist irbesartan with amlodipine or placebo for nephroprotective effects, showed that the mean BP at visits following randomization was 140/77 mmHg in the irbesartan group, 141/77 mmHg in the amlodipine group, and 144/80 mmHg in the placebo group. The mean arterial pressure was significantly higher (by 3.3 mmHg) in the placebo group than in the two active treatment groups (*p* = 0.001 for both comparisons); the active treatment groups did not differ significantly. The distribution of classes of non-study drugs used to control BP—primarily diuretics, β-adrenergic receptor antagonists, α-adrenergic receptor antagonists, and central α_2_ agonists—was similar in all groups. Patients in the placebo group required an average of 3.3 non-study drugs for the BP control, compared with an average of 3.0 among the patients in the irbesartan and amlodipine groups. The antihypertensive effect of irbesartan was comparable to amlodipine. Still, the former was more potent in nephroprotection (the serum creatine concentration increased more slowly, and a more significant reduction of proteinuria was observed in the irbesartan group than in amlodipine) [128]. The antihypertensive efficacy of other ARB in T2D patients, i.e., olmesartan [139] and candesartan [140], was also documented.

The *multicenter double-blind, randomized Bergamo Nephrologic Diabetes Complications Trial* (BENEDICT) was designed to assess whether ACEI and non-dihydropyridine calcium-channel blocker (CCB), alone or in combination, prevent microalbuminuria in subjects with hypertension, T2D, and normal urinary albumin excretion. Throughout the study, the average trough SBP was 139 ± 10 mmHg, and the average trough DBP was 80 ± 6 mmHg in the group receiving trandolapril plus verapamil; the corresponding values were 139 ± 12 mmHg and 81 ± 6 mmHg in the trandolapril group; 141 ± 10 mmHg and 82 ± 6 mmHg in the verapamil group; and 142 ± 12 mmHg and 83 ± 6 mmHg in the placebo group. The difference was significant (*p* < 0.002) for systolic and diastolic BP between either the trandolapril-plus-verapamil group or the trandolapril-alone group and the placebo group, but the results were not significantly different for the verapamil group as compared with the placebo group. The antiproteinuric properties of trandolapril were also observed. The authors concluded that in subjects with T2D and arterial hypertension, normoalbuminuria, and normal renal function, therapy with trandolapril plus verapamil or trandolapril alone prevented the onset of albuminuria. The effect of ACEI did not appear to be enhanced by adding a non-dihydropyridine CCB. These findings suggest that in hypertensive T2D patients with normal renal function, an ACEI may be a valuable medication for controlling BP [141].

Additionally, the five-year study showed a more pronounced antihypertensive effect of telmisartan (80 mg daily) than enalapril (20 mg daily) in T2D patients. The mean reduction in SBP with telmisartan was 6.9 mmHg, compared with 2.9 mmHg with enalapril (95 percent confidence interval, –8.5 to 0.5 mm Hg). At the end of the study, 75 percent of the subjects had an SBP of less than 160 mmHg, and 42 percent had an SBP of less than 140 mmHg; there was no significant difference between these groups in this respect, and telmisartan did not prove inferior to enalapril in providing long-term nephroprotection [142]. In the *Ongoing Telmisartan Alone and in Combination With Ramipril Global End Point Trial* (ONTARGET), both RASi (ramipril or telmisartan alone and their combination) significantly reduced BP at 6 weeks: 6.4/4.3 mmHg in the ramipril group, 7.4/5.0 mmHg in the telmisartan group, and 9.8/6.3 mmHg in the combination-therapy group. Patients in the telmisartan and the combination-therapy groups continued to have slightly lower BP throughout the study period (average reductions, 0.9/0.6 mmHg and 2.4/1.4 mmHg, respectively) compared to patients in the ramipril group [143]. Still, the combination therapy with ARB and ACEI is now not recommended due to side effects [143].

Therefore, both ARBs and ACEIs exert antihypertensive and, to some extent, nephroprotective properties in hypertensive T2D patients. Due to the more significant number of studies with ARB confirming its efficacy and its slightly stronger antihypertensive and nephroprotective effects in comparison with ACEI in this population, we suggest using the ARBs (particularly losartan and irbesartan) with well-documented nephroprotective impact in T2D patients (see in Statement 4.1.1) as a first choice in antihypertensive treatment in T2D patients with CKD. Moreover, RASi should be given at the maximum tolerated doses to achieve optimal nephroprotection [135,143,144]. At the same time, the dual combination two of RASi should be avoided [145] (see in Statement 4.2).

It should be kept in mind that RAS blockade in CKD may cause hyperkalemia or kidney function deterioration, especially in patients with eGFR < 30 mL/min/1.73 m^2^ thus, monitoring serum potassium and creatinine during ARB or ACEI therapy is mandatory (though the exact frequency of such testing has not been yet established; we suggest to measure serum potassium and creatinine at least 7–10 days after initiation or intensification of RAS blockade). It is discussed in detail in Statement 3.6.

### 3.4. Statement 3.4

#### 3.4.1. Statement 3.4.1

A combined antihypertensive therapy should be used in most hypertensive patients with CKD and T2D [1A].

- *Comment to Statement 3.4.1* 

Considering the difficulties of BP control in diabetes and CKD patients and the importance of achieving BP targets in patients with high CV risk [19,28], antihypertensive therapy should be initiated with dual combination therapy in most patients. Only in patients with hypertension with BP less than 10 mmHg over the blood pressure target (i.e., with SBP between 140 and 150 mmHg) might antihypertensive therapy initiation with single agents (preferably ARB or ACEI) be appropriate.

#### 3.4.2. Statement 3.4.2

We suggest adding dihydropyridine calcium channel blocker (CCB) and/or a thiazide-like or thiazide diuretic to RASi [1B]. In patients with eGFR > 30 mL/min/1.73 m^2^, all thiazide-like or thiazide diuretics might be used; in patients with eGFR ≤ 30 mL/min/1.73 m^2^, chlorthalidone should be preferably used [1B]. We suggest monitoring serum sodium and potassium concentrations in patients treated with diuretics [expert opinion].

- *Comment to Statement 3.4.2* 

Considering the high CV risk and resistant hypertension in patients with T2D, the majority of them should be treated at least with dual antihypertensive therapy. According to the data from clinical trials, dihydropyridine calcium channel blockers (CCB) are effective as an antihypertensive management tool in this population. The *Fosinopril Versus Amlodipine Cardiovascular Events Randomized Trial* (FACET) study compared the effects of fosinopril and amlodipine in the T2D patients with hypertension and serum creatinine < 1.5 mg/dL over the 2.5 years of follow-up. Both treatments significantly decreased SBP and DBP, but a more significant reduction was observed in patients treated with amlodipine than in those treated with fosinopril (−19 and −13 mmHg, respectively). The two treatment groups had the same decrease in DBP compared with baseline (−8 mmHg) [146].

In the *Avoiding Cardiovascular Events through Combination Therapy in Patients Living with Systolic Hypertension* (ACCOMPLISH) trial, which assessed CV events in patients (also including patients with diabetes) who received treatment with either benazepril plus amlodipine or benazepril plus hydrochlorothiazide, the mean BP after dose adjustment in a follow-up of 35 months was 131.6/73.3 mmHg in the benazepril–amlodipine group and 132.5/74.4 mmHg in the benazepril–hydrochlorothiazide group. The mean difference between these two groups was 0.9 mm Hg in SBP and 1.1 mm Hg in DBP (*p* < 0.001 for both systolic and diastolic pressures). The benazepril–amlodipine combination was superior to the benazepril–hydrochlorothiazide combination in reducing CV events in patients with hypertension who were at high risk of such events [147]. Consequently, combining RASi with CCB or diuretics is an excellent first-line therapy for managing BP in CKD. The possibility of effectively controlling BP, especially in advanced CKD, without a diuretic is low, and additionally, diuretics are also frequently needed due to the high prevalence of fluid overload and sodium sensitivity of hypertension. It is a consequence of the expansion of the extracellular fluid because of a decreased capacity of the kidneys in CKD to excrete sodium [148].

Thiazide-like diuretics, or thiazides, have been recommended as the preferred diuretics in the treatment of hypertension [136,149]. Such a preference is based on the results of several randomized, placebo-controlled studies with these agents (also comprising patients with T2D) [150,151,152,153,154]. In the ADVANCE trial, the combination of perindopril and indapamide treatment in hypertensive T2D patients decreased SBP of 5.6 mmHg and DBP of 2.2 mmHg during 4.3 years of follow-up. A significant 18% reduction of CV death was observed in the active group vs. placebo (3.8% vs. 4.6%; 0.82, 0.68–0.98, *p* = 0.03). Moreover, the perindopril and indapamide treatment showed the nephroprotective efficacy: 21% reduction in all renal events (95% CI 15–27%, *p* < 0.0001), with a borderline significant reduction in new onset or worsening nephropathy (development of albuminuria, doubling of S_cr_ to at least 200 μmol/L, need for renal replacement therapy, or death due to renal disease) 3.3% vs. 3.9% (relative risk reduction 18% [–1 to 32%], *p* = 0.055) and a significant reduction in the development of albuminuria (19.6% vs. 23.6%; 21% [14–27%]; *p* < 0.0001) [153].

In the past, thiazide-like diuretics or thiazides were considered ineffective in advanced CKD, but recent data indicate that thiazide-like or thiazide diuretics may benefit BP control in more advanced CKD [154,155,156]. In one of the studies, furosemide (60 mg) and hydrochlorothiazide (HCTZ, 25 mg) were compared in 23 patients with hypertension and CKD stages 4 or 5 (including subjects with T2D). HCTZ was as effective as furosemide in reducing BP, and combining thiazide with the loop diuretic synergized BP lowering [155]. It should be noted that thiazide diuretics such as hydrochlorothiazide may slightly elevate fasting blood glucose and blood HbA1c concentrations. However, this effect is small, and the benefits of antihypertensive effectiveness are generally thought to outweigh these side effects [157].

In a recent *Chlorthalidone in Chronic Kidney Disease* (CLICK) study, the efficacy of chlorthalidone was evaluated in patients with CKD with mean eGFR 23.2 mL/min/1.73 m^2^ and poorly controlled hypertension, among whom 76% suffered T2D. Chlorthalidone therapy improved BP control (reduction of the 24 h ambulatory SBP to −10.5 mmHg and DBP to −3.9 mmHg in the chlortalidone group was observed) at 12 weeks and reduced albuminuria and proteinuria as compared to placebo [158]. In the chlorthalidone group, the reduction in the eGFR was more pronounced than in the placebo group after initiation of the assigned regimen (change from baseline in the eGFR was −2.7 mL/min/1.73 m^2^ in the chlorthalidone group vs. without changes in the placebo group at four weeks after the treatment initiation). This difference was attributed to the potent BP reduction, presumably leading to beneficial attenuation of hyperfiltration. Still, two weeks after discontinuing the assigned trial regimen, the eGFR was similar in the two groups, documenting the hemodynamic nature of this phenomenon. Results of a recent meta-analysis, including, among others, data from the CLICK study, demonstrated that thiazide-like or thiazide diuretics (hydrochlorothiazide, chlortalidone, and butizide) in patients with eGFR 13–27 mL/min/1.73 m^2^ are effective antihypertensive agents [159]. It should be mentioned that higher thiazide doses are necessary to achieve a therapeutic effect in CKD because these drugs act on the luminal side of the tubular epithelium and must be delivered to the tubules by peritubular capillaries and then excreted [160].

It should be remembered that patients receiving a thiazide or thiazide-like diuretic may develop electrolyte disturbances with hyponatremia (which may be severe and life-threatening) or hypokalemia, which is a risk factor for life-threatening cardiac arrhythmias [161,162]. Notably, the subjects with a genetic deficiency in the prostaglandin transporter activity are in the risk group for thiazide-induced hyponatremia [163]. Therefore, the patients for whom such treatment has been prescribed require regular laboratory monitoring of serum sodium and potassium concentrations (at least 2–3 weeks after initiation or dose escalation, and then at least annually, but preferably on every occasion of diagnostic blood sampling).

### 3.5. Statement 3.5

In CKD patients with T2D, resistant hypertension, and no tendency towards hyperkalemia (i.e., current serum potassium concentration ≤ 4.5 mmol/L and lack of clinically meaningful, symptomatic hyperkaliemia incident in the past), we suggest using steroidal mineralocorticoid antagonist (MRA)—spironolactone [2B].

- *Comment to Statement 3.5* 

Due to the frequent occurrence of resistant hypertension among CKD patients, particularly with T2D, triple antihypertensive drug therapy may not control BP, and the addition of spironolactone to the standard antihypertensive treatment may effectively regulate BP and reduce albuminuria [164]. The meta-analysis of Hou et al. showed that the addition of spironolactone to conventional antihypertensive treatment, including diuretics and optimal doses of an ACEi or ARB in patients with diabetic nephropathy, reduced albuminuria by 33% (95% CI 25–41) (*p*< 0.001), fractional clearance of albumin by 40% (24–53) (*p* < 0.001), and BP by 6 mmHg (2–10) for SBP and 4 mmHg (2–6) for DBP (*p* < 0.001 for both) [164]. Moreover, spironolactone may delay CKD progression in T2D subjects [165].

The PATHWAY-2 study was a randomized controlled trial to compare spironolactone with other BP-lowering drug treatments in a population of patients with resistant hypertension, including T2D patients (14% were diabetic); however, with the exclusion of patients with eGFR < 45 mL/min/1.73 m^2^ and baseline potassium above 4.5 mmol/L. Enrolled T2D patients had SBP ≥ 135 mmHg despite the treatment with maximum tolerated doses of three drugs: ACEI or an ARB, a CCB, and diuretic for at least 3 months. After a one-month single-blind placebo run-in, patients rotated through four 12-week cycles of once-daily oral treatment with spironolactone 25–50 mg, doxazosin modified release 4–8 mg, bisoprolol 5–10 mg, and placebo. The average reduction in home SBP throughout the treatment cycle with spironolactone was superior to each of the comparators. The SBP reduction in the spironolactone group was higher than in the placebo (−8.70 mmHg [95% CI −9.72 to −7.69]; *p* < 0.0001); also in the other two active treatments (doxazosin and bisoprolol, −4.26 [−5.13 to 3.38]; *p* < 0.0001); and each of the other individual treatments, doxazosin (−4.03 [−5.04 to 3.02]; *p* < 0.0001) and bisoprolol (−4.48 [−5.50 to –3.46]; *p* < 0.0001). Notably, in this trial, the need to study drug discontinuation due to renal impairment, hyperkaliemia, and gynecomastia was not more frequent in spironolactone-treated patients as compared to other active treatments and placebo. Bisoprolol and doxazosin were more effective than placebo at reducing BP as an add-on therapy for resistant hypertension but significantly less effective than spironolactone [166].

Spironolactone reduces proteinuria and SBP in adults with mild-to-moderate CKD but may increase the risk of hyperkaliemia and AKI (particularly when added to ACEi or ARB) and gynecomastia [167]. On the other hand, spironolactone may prevent diuretic-induced hypokalemia and is recommended in the treatment of heart failure. However, careful monitoring of serum potassium concentration is mandatory in CKD during spironolactone treatment. Hyperkaliemia is the critical limitation of the widespread use of MRA in patients with advanced CKD. Due to the risk of hyperkalemia, it should not be started in patients with serum potassium > 4.5 mmol/L or clinically meaningful (i.e., symptomatic) hyperkaliemia incidents in the past [154].

The alternatives to spironolactone include eplerenone and finerenone. Due to its less potent BP-lowering properties and lack of approval in this indication, eplerenone should not be recommended to treat hypertension in most CKD patients. Moreover, the antiproteinuric properties of eplerenone remain less documented than those of spironolactone [168]. Finerenone, a novel selective non-steroidal MRA, is characterized by low antihypertensive potential. Therefore, it is not used in the treatment of resistant hypertension. Finerenone reduces the risk of kidney function decline and CV events in patients with CKD and T2D [169,170]. It is discussed in detail in Statement 4.3.2.

### 3.6. Statement 3.6

In case of hyperkalemia in the range of 5.0–5.5 mmol/L after initiating ARB or ACEi and/or spironolactone or finerenone or increasing their doses, we suggest using methods that reduce serum potassium concentration other than ARB or ACEI or spironolactone or finerenone dose reduction or withdrawal (such as dietary measures, thiazide, thiazide-like or loop diuretics, treatment of metabolic acidosis, agents that reduce potassium absorption in the gastrointestinal tract approved for chronic use, i.e., patiromer, and sodium zirconium cyclosilicate). In case of hyperkalemia with serum potassium concentration > 5.5 mmol/L after initiating ARB or ACEI and/or spironolactone or finerenone or following their dose increases, we suggest dose reduction or discontinuation of these agents, further monitoring of serum potassium concentration, and a possible resumption of ARB or ACEI and/or spironolactone or finerenone treatment at a lower dose with subsequent dose increase to maximum tolerated in combination with the measures mentioned above to lower serum potassium concentration [expert opinion].

- *Comment to Statement 3.6* 

Hyperkalemia is a frequent complication of treatment with RASi and spironolactone or, to a lesser extent, finerenone, mainly in patients with advanced CKD [171,172]. Because of the efficacy of these drugs in the management of CKD patients and their beneficial effects on BP, CV morbidity, and progression of kidney failure, the prevention of hyperkalemia should be recommended as a first line. First of all, a diet with a moderate intake of potassium-rich foods and the elimination of potassium-containing salt substitutes should be recommended, as should discontinuation of any drugs that can impair kidney excretion of potassium (among them over-the-counter nonsteroidal anti-inflammatory drugs and trimethoprim). Dietary supplements and herbal preparations should be avoided. General measures to prevent constipation should include sufficient fluid intake, exercise, and possibly laxatives. In the next step, diuretics and SGLT2i to enhance the renal excretion of potassium should be introduced. However, it should be remembered that too-aggressive therapy with diuretics can lead to AKI and electrolyte abnormalities. Hypokalemic response to diuretics is diminished with low eGFR and depends on the type of diuretic used [32]. SGLT2i generally prevents the excess risk of hyperkalemia in patients with T2D and/or CKD [44,45]. Diuretics are most effective for hyperkalemia management when there is simultaneous volume overload and hypertension. Metabolic acidosis increases hyperkalemia risk in CKD, and the treatment with oral sodium bicarbonate in CKD patients with metabolic acidosis is an effective strategy for minimizing the risk of this abnormality [173,174]. The recommendations for managing metabolic acidosis are presented in Part 4 of the current paper and more extensively in the statement of the Working Group of the Polish Society of Nephrology [173].

In the AMBER trial, spironolactone with the addition of placebo or patiromer in patients with treatment-resistant hypertension and eGFR between 25 and 45 mL/min/1.73 m^2^ effectively reduced BP, but the rates of hyperkalemia (potassium 5.5 mmol/L) were at about 60 and 35%, respectively, at 12 weeks [174]. Thus, the use of spironolactone as an antihypertensive agent in CKD patients with resistant hypertension should be restricted to patients without a tendency towards hyperkalemia (i.e., current serum potassium concentration ≤ 4.5 mmol/L and lack of clinically significant, symptomatic hyperkaliemia incident in the past) only. The use of prevention hyperkalemia measures mentioned above (including patiromer) is advisable to maintain serum potassium below 5.0 mmol/L [175,176].

### 3.7. Statement 3.7

In CKD patients with T2D and resistant hypertension with a tendency to hyperkaliemia (i.e., serum potassium concentration > 4.5 mmol/L or clinically meaningful, symptomatic hyperkaliemia incident in the past) or other contraindications to spironolactone, other antihypertensive drugs should be added to achieve target office BP: doxazosin, central α_1_-receptor agonists, β-adrenergic antagonists (in patients with no competing indications, nebivolol, bisoprolol or carvedilol should be preferred) and loop diuretics [expert opinion].

- *Comment to Statement 3.7* 

As we mentioned above, bisoprolol and doxazosin are effective in reducing BP as add-on therapy for resistant hypertension but significantly less effective than spironolactone [166]. Some small studies demonstrated the efficacy of α-adrenergic receptor antagonists, such as doxazosin, in treating hypertension in patients with diabetic kidney disease [177,178,179]. Their pharmacokinetic profile is independent of kidney function and metabolically neutral. Moreover, it was demonstrated that doxazosin may decrease insulin resistance [180]. However, the use of these agents is associated with the risk of orthostatic hypotension, which should be kept in mind mainly in the case of older patients with CKD.

Centrally-acting drugs, such as clonidine or methyldopa, are generally considered safe in patients with CKD [181]. Still, there is a lack of studies assessing their efficacy in patients with CKD and T2D, except for methyldopa in hypertensive pregnant women with diabetes and nephropathy [182]. They can be used in patients with resistant hypertension or when other BP-lowering medications are contraindicated. In patients with resistant hypertension and normal renal function, clonidine was as effective as spironolactone in lowering BP [183]. One small study that enrolled 29 hypertensive diabetic patients showed the efficacy of rilmenidine in BP normalization but without impact on proteinuria [184].

**Figure 1:**
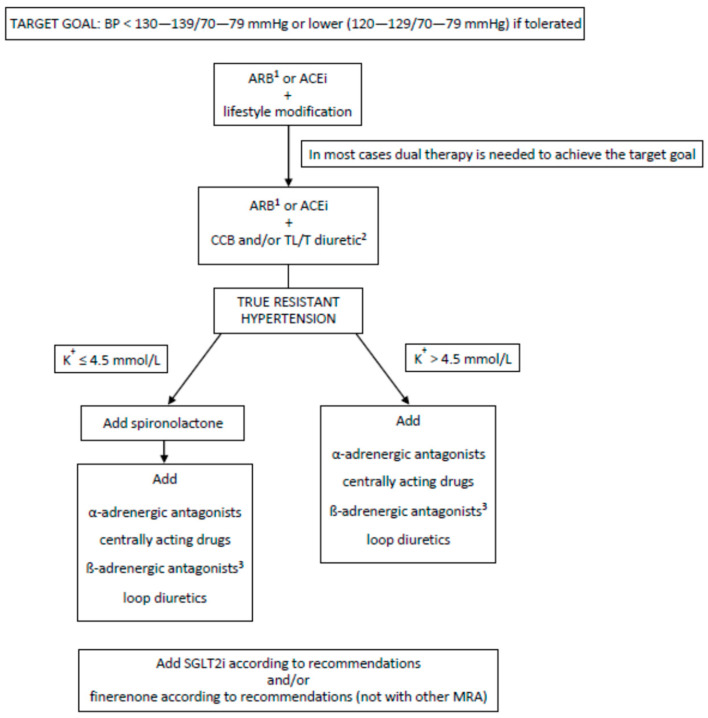
Blood pressure-lowering therapy in chronic kidney disease patients with type 2 diabetes. ARB: angiotensin receptor blockers; ACEi: angiotensin-converting enzyme inhibitors; CCB: dihydropyridine calcium channel blockers; MRA mineralocorticoid steroidal antagonist; TL/T diuretic: thiazide-like or thiazide diuretics; ^1^ losartan and irbesartan are preferred; ^2^ chlorthalidone in patients with eGFR < 30 mL/min/1.73 m^2^, ^3^ bisoprolol, nebivolol and carvedilol are preferred; SGLT2i—sodium–glucose transporter 2 inhibitor; MRA—mineralocorticoid receptor antagonist.

It has been established that β-adrenergic antagonists reduce all-cause mortality in T2D patients with heart failure and reduced ejection fraction, patients with arrhythmia, hypertrophic cardiomyopathy, or coronary heart disease [185,186,187]. Among the β-adrenergic antagonists, nebivolol with vasodilating properties, bisoprolol, a highly β_1_-selective adrenergic antagonist, and carvedilol β-adrenergic with vasodilating properties due to α_1_-blockade [186] are considered helpful as adjunctive drugs in hypertension treatment. The use of β-adrenergic antagonists improves outcomes in T2D patients with heart failure. It should be recommended in this population, with a preference for agents such as carvedilol and nebivolol because of their ability to improve insulin sensitivity and no adverse effects on glycemic control [186]. RCTs with carvedilol, bisoprolol, metoprolol, and nebivolol showed improved outcomes in patients with heart failure (including those with diabetes) [186]. It should be kept in mind that bradycardia is a well-described side effect of this drug group, mainly in patients with CKD. The *Glycemic Effects in Diabetes Mellitus: Carvedilol-Metoprolol Comparison in Hypertensives* (GEMINI) trial involved patients with T2D and hypertension. It compared the metabolic and glycemic effects of treatment with metoprolol tartrate to treatment with carvedilol. Carvedilol did not affect glycemic control and improved insulin sensitivity [187]. Taking the above into consideration, carvedilol, bisoprolol, and nebivolol may be regarded as the preferred drugs among β-adrenergic antagonists in the treatment of hypertension in T2D patients, especially at high CV risk. Loop diuretics can also be used as an element of add-on therapy for resistant hypertension. Moreover, loop diuretics must be used in CKD patients with signs of overhydration in advanced CKD (usually with eGFR < 30 mL/min/1.73 m^2^), concomitant heart failure, or liver cirrhosis. It should be stressed that, in contrast to thiazide or thiazide-like diuretics, there are no studies documenting improvement of cardiovascular morbidity with these agents. In comparison to furosemide, torsemide may be more useful in the treatment of hypertension because of its longer half-life, which is also an advantage because it can be dosed once daily [168,188]. Blood pressure-lowering pharmacotherapy in CKD with T2D patients was summarized in Figure 1.

## 4. Inhibition of Renin–Angiotensin–Aldosterone Axis

### 4.1. Statement 4.1

We recommend using a RASi (ARB or ACEi) (if not contraindicated) in patients with CKD and T2D with proteinuria or urinary albumin/creatinine ratio ≥ 300 mg/g [1A] or urinary albumin/creatinine ratio ≥ 30 mg/g [2C], and we suggest using a RASi (if not contraindicated) in CKD and T2D patients with urinary albumin/creatinine ratio < 30 mg/g [expert opinion].

- *Comment to Statement 4.1* 

The recommendation of using RASi in patients with severely increased albuminuria is based on evidence from two randomized, controlled clinical trials that demonstrated a beneficial effect of ARB on renal prognosis, independent of the antihypertensive effect. These studies compared losartan (50–100 mg) with placebo in *The Reduction of Endpoints in NIDDM with the Angiotensin II Antagonist Losartan Study* (RENAAL) and irbesartan (300 mg) with placebo or calcium channel blockers in the *Irbesartan Diabetic Nephropathy Trial* (IDNT), respectively [128,189]. Both studies included patients with T2D and severely increased albuminuria: IDNT (proteinuria ≥ 900 mg/24 h and a S_cr_ of 1 to 3 mg/dL), RENAAL (urine protein/creatinine ratio ≥ 300 mg/g and a serum creatinine concentration of 1.3 to 3 mg/dL). The RENAAL study included patients with and without hypertension, while the IDNT study encompassed only patients with hypertension. The use of irbesartan in the IDNT trial resulted in a 20% reduction in the risk of the composite endpoint (S_cr_ doubling, development of ESRD, all-cause mortality) compared to placebo and a 23% reduction in this risk compared to amlodipine at 2.6 years, which was independent of blood pressure [116]. Losartan in the RENAAL study reduced the incidence of a doubling of S_cr_ by 25% and ESRD by 28% at 3.4 years compared to placebo. The renoprotective effect conferred by losartan also exceeded the effect attributable to the small differences in blood pressure between the treatment groups [190].

We also recommend using RASi (ARB or ACEi) in patients with T2D and moderately increased albuminuria, i.e., urinary albumin/creatinine ratio ≥ 30 mg/g. The overall quality of this evidence is rated as moderate, given that it has been driven by the outcome of the albuminuria lowering only, not by the doubling of baseline S_cr,_ i.e., CKD progression. *The Irbesartan in Type 2 Diabetes With Microalbuminuria 2* (IRMA-2) and the *Incipient to Overt: Angiotensin II Blocker, Telmisartan, Investigation on Type 2 Diabetic Nephropathy* (INNOVATION) trials were placebo-controlled studies enrolling patients with moderately increased albuminuria (30–300 mg/g creatinine) [190,191] designed to find whether RASi reduced the risk of development of severely increased albuminuria (>300 mg/g creatinine). The IRMA-2 trial demonstrated that irbesartan, in a dose-dependent manner, reduced the risk of albuminuria increase. This effect was independent of the blood pressure-lowering properties of irbesartan [192]. Similarly, in the INNOVATION trial, telmisartan, in both doses of 40 mg and 80 mg, reduced the risk of albuminuria increase as compared to placebo after 1 year of follow-up [191]. The beneficial effect of telmisartan in delaying albuminuria increased from moderate to severe and persisted after adjustment for the difference in BP values between the placebo and active treatment groups [191].

The renal benefits of RASi in patients with T2D and albuminuria but without hypertension have not been specifically studied, and some doubt whether it is worth using RASi in such patients [192]. It must be taken into account that 3.5% of patients in the RENAAL trial and 30.9% of subjects in the INNOVATION trial had normal BP, which may suggest that ARB use may be beneficial in normotensive patients as well [193,194]. Given that albuminuria is a strong predictor of CKD progression in this population and RASi significantly reduce albuminuria [195,196], we suggest using a RASi also in patients without hypertension but with SBP not lower than 120 mm Hg.

There is no evidence to support that either ACEi or ARB has a beneficial effect on renal prognosis in people with T2D and normal ACR (i.e., <30 mg/g). Several studies analyzed the effect of RASi on preventing the transition from normoalbuminuria to moderately increased albuminuria. In the *Bergamo Nephrologic Diabetes Complications Trial* (BENEDICT) study, trandolapril and ARB prevented the development of albuminuria, and the effect seemed to be independent of blood pressure reduction [141]. Furthermore, in the *Randomized Olmesartan and Diabetes Microalbuminuria Prevention* (ROADMAP), the use of ARB olmesartan was associated with a delayed onset of albuminuria, even though BP control in both groups was excellent according to current standards [139]. The potential nephroprotective effect of RASi in patients with T2D and normal ACR ratio has also been analyzed in several meta-analyses. In the meta-analysis of 6 studies, including 16,921 normoalbuminuric patients, a 16% RR reduction for the development of albuminuria in the ACEi/ARB treatment group as compared to placebo groups was found [183]. In the pooled analysis of sixteen trials (7603 normoalbuminuric patients with diabetes), ACEi significantly reduced the onset of albuminuria compared to placebo (six trials, 3840 patients; RR 0.60; 95% CI 0.43 to 0.84) and to calcium antagonists (four trials, 1210 patients; RR 0.58; 95% CI 0.40 to 0.84) [195]. Given also studies demonstrating non-hemodynamic beneficial effects of ACEi and ARB, such as attenuation of local inflammation and fibrosis [193], it is reasonable to expect that RAS blockade may be an effective therapeutic option in normoalbuminuric patients, but the strength of this recommendation is very weak at present.

#### 4.1.1. Statement 4.1.1

ARB should be considered as the preferred therapeutic option [1A], and ACEi may be used in the case of ARB intolerance [2C]. We recommend using losartan and irbesartan as preferred nephroprotective agents [1A], but it is possible to use other ARBs [2D].

- *Comment to Statement 4.1.1* 

Despite differences in the mechanism of action, experimental and clinical studies revealed that ARB and ACEi produce similar improvements in glomerular hemodynamics and have similar effects on the major determinants of CKD progression, namely BP and proteinuria [197,198]. Only a few long-term head-to-head studies have been designed to compare the effects of ARB and ACEi on the progression of diabetic kidney disease. For instance, the *Diabetics Exposed to Telmisartan and Enalapril* (DETAIL) study with a follow-up of five years found that treatment with ARB telmisartan (80 mg daily) or ACEi enalapril (20 mg daily) similarly decreased blood pressure and albuminuria and reduced the rate of eGFR decline in 250 patients with T2D and early-stage CKD (82% moderately increased albuminuria and 18% severely increased albuminuria to a maximum of 1.4 g/d and a baseline eGFR of approximately 93 mL/min/1.73 m^2^ [142]. Therefore, we do not believe the evidence is sufficiently robust to demonstrate that ARB is better than ACEi. Nevertheless, ARB should be considered as the preferred therapeutic option given that their nephroprotective potential was evidenced in randomized controlled trials. We recommend using ARBs, whose beneficial effects on renal outcomes have been proven, i.e., irbesartan and losartan [131,194]. At the same time, we take the position that this protective effect is not due to the specific action of these preparations but to the impact of the entire class of ARB. Hence, we believe it is also correct to use other ARB preparations than those recommended above.

#### 4.1.2. Statement 4.1.2

We recommend using ARB or ACEi in maximally tolerated doses (according to individual tolerance and summaries of product characteristics with possible reduction accordingly with eGFR decline) [2C]. Doses of ARB or ACEi should be carefully up-titrated with their tolerance monitoring [expert opinion].

- *Comment to Statement 4.1.2* 

We judge that ARB or ACEi should be titrated to the maximally tolerated doses approved by regulatory agencies/respective summaries of product characteristics, mainly because the renal benefits were achieved in trials when using such doses [129,190]. Furthermore, evidence exists that inhibition of the RAS is a dose-related phenomenon [199]. Enhancing the RAS inhibition by increasing the dosage of ARB or ACEi allows for a more significant decrement in proteinuria [191]. What is more, post hoc analyses of randomized controlled trials and observational cohort trials have demonstrated that larger initial albuminuria reduction is associated with better long-term outcomes [199,200].

It was also speculated that a more aggressive RAS blockade by using a single ACEi or ARB in ultra-high doses could reduce further proteinuria [201]. Some exploratory clinical studies conducted in small populations seemed to support this hypothesis. *The Supra Maximal Atacand Renal Trial* (SMART) was designed to compare the effects of candesartan in supramaximal dosage and the highest approved antihypertensive dosage in patients with mixed CKD (eGFR > 30 mL/min/1.73 m^2^) and persistent proteinuria ≥ 1 g/24 h [140]. More than 50% of the patients included in the study had a diagnosis of T2D. The mean difference in the percentage change in proteinuria for patients receiving daily 128 mg candesartan compared with those receiving daily 6 mg/d candesartan was −33.05% (95% confidence interval −45.70 to −17.44; *p* < 0.0001). Reductions in blood pressure were not different across the treatment groups. However, as long as such management has not been tested in large clinical trials with long-term follow-up, the use of doses exceeding the maximal approved by regulatory agencies should not be used.

#### 4.1.3. Statement 4.1.3

We recommend continuing ARB or ACEi also in advanced CKD (patients with eGFR < 30 mL/min/1.73 m^2^) and T2D [1B]. We recommend reducing the dose or preferably temporarily only, discontinuing ARB or ACEi in symptomatic hypotension or, despite medical treatment, uncontrolled hyperkalemia (see also Statement 3.6) [expert opinion].

- *Comment to Statement 4.1.3* 

A small observational study showing some improvement in eGFR after stopping RASi led to the hypothesis that continuing these drugs in patients with advanced CKD (46% with diabetes) might accelerate the need for renal replacement therapy [202]. Furthermore, a recent large, real-world observational study from Sweden of 10,254 patients with advanced CKD (49.5% with diabetes) on RASi and under routine care by nephrologists showed that discontinuing this treatment is associated with a decrease in absolute risk of initiating renal replacement therapy [203]. On the contrary, in the meta-analysis of Nistor et al. [204], continuing treatment with RASi, either ACEi or ARB, has favorable effects on renal outcomes in patients with diabetes and CKD stages 3–5, resulting in a 22% reduction in the risk for the composite outcome of need for renal replacement therapy or S_cr_ doubling. Similarly, the post hoc, secondary analysis of the RENAAL trial showed the renoprotective effect exerted by losartan; ARB was independent of the severity of renal insufficiency and uniformly decreased the risk of ESRD also in patients in the subgroup with the most advanced CKD (the highest tertile of baseline S_cr_, i.e., S_cr_ between 2.1 and 3.6 mg/dL) [205].

The randomized, open-label STOP-ACEi trial was designed to ultimately determine whether ACEi or ARB discontinuation could slow CKD progression in patients with stage 4–5 CKD. Four hundred eleven patients with diabetic and non-diabetic CKD were randomly assigned to discontinue or continue RASi. A total of 32.4% of the patients included in the study were diagnosed with T2D. At three years, the least-squares mean (±SE) eGFR was 12.6 ± 0.7 mL/min/1.73 m^2^ in the discontinuation group and 13.3 ± 0.6 mL/min/1.73 m^2^ in the continuation group (difference, −0.7; 95% confidence interval [CI], −2.5 to 1.0; *p* = 0.42). ESRD, or the initiation of renal replacement therapy, occurred in 128 patients (62%) in the discontinuation group and 115 patients (56%) in the continuation group [19]. Despite some apparent limitations (open-label nature, no dosing information), the STOP-ACEi trail evidenced that discontinuing RASi in patients with advanced CKD does not improve kidney function (although adverse CV effects were not observed either) [206]. Taking into account the results of the above-mentioned studies, it may be concluded that the decision to continue or discontinue RASi should be made in the context of the individual patient’s clinical presentation, blood pressure control, and treatment tolerability.

#### 4.1.4. Statement 4.1.4

We suggest that ARB or ACEi for nephroprotection should be accompanied by dietary salt intake restriction [2C].

- *Comment to Statement 4.1.4* 

Hypertension is a frequent finding in CKD patients and is considered, among others, a consequence of sodium sensitivity. As we already mentioned in this document, a reduction in dietary sodium intake induces improved BP control, and such an approach may reduce the need for adding antihypertensive medications and/or escalating their doses. No data from adequately designed RCTs exist to evaluate the effect of a low-sodium diet on clinically meaningful renal outcomes in patients with CKD and diabetes. However, the results of exploratory and observational studies indicate that this may be the case. In the *Chronic Renal Insufficiency Cohort* (CRIC) study, a large observational study carried out in 3757 CKD patients (47.7% with diabetes) followed for almost seven years, high sodium excretion exceeding 4476 mg/24 h was associated with a higher risk of CKD progression as compared to the group with the low sodium excretion (less than 2686 mg/24 h). This association was independent of other essential variables modifying CKD progression rate, including RASi and other antihypertensive medications [207]. It agrees with a meta-analysis reporting that in patients with diabetic and non-diabetic CKD on a low-salt diet, RASi had an augmented antiproteinuric effect. In the pooled analysis of eleven studies with 516 participants and follow-ups ranging from 1 to 6 weeks, an average reduction in sodium intake by 92 mmol/d (5.4 g salt) was associated with a 41.9% (95% confidence interval, −56.4 to −27.4%) reduction in urinary albumin excretion in patients on concomitant RASi [28]. Furthermore, in a post hoc analysis of 1177 patients with T2D included in the RENAAL and the IDTN trials, high urinary sodium excretion was associated with an increased risk of renal outcomes in patients treated with ARB but not patients treated with non-RASi-based antihypertensive therapy [208]. The synergic effect of low sodium intake and RAS inhibition may be due to the enhanced angiotensin-converting enzyme activity and increased angiotensin II type 1 receptor density in renal tissue triggered by high salt intake [209], counteracting the effect of RASi on glomerular hemodynamics and proteinuria.

#### 4.1.5. Statement 4.1.5

We suggest measuring serum creatinine and potassium concentrations within 7–14 days after initiating ARB or ACEi therapy and increasing the dose of ARB or ACEi [expert opinion]. If serum creatinine concentration rises less than 30% from baseline after initiating ARB or ACEi therapy or increasing their doses, we suggest continuing an ARB or ACEi. For patients with an increase of 30% and more of baseline, we suggest discontinuing ARB or ACEi and evaluating the causes of serum creatinine concentration increase (including diagnostic procedures to detect renal artery stenosis) [expert opinion].

- *Comment to Statement 4.1.5* 

ACEi and ARB are potent antihypertensive drugs that counteract the vasoconstrictor effect of angiotensin II. Precisely, they cause more significant dilatation of the efferent glomerular arterioles selectively, resulting in an acute decrease in intraglomerular pressure, a reduction of glomerular filtration, and a possible increase in S_cr_ shortly after initiation of the therapy. An increase in S_cr_, if it occurs, will typically happen during the first 2 weeks of treatment initiation, and it should stabilize within 2 to 4 weeks in the setting of normal sodium and fluid intake. A review of 12 randomized controlled trials that evaluated CKD progression demonstrated a strong association between acute increases of S_cr_ of up to 30% from baseline that stabilized within 2 months of ACEi therapy initiation and long-term preservation of kidney function [210]. This remains in line with the RENAAL investigators who concluded that acute change (“acute dip”) in GFR in losartan-treated patients, but not placebo-treated patients, was associated with a reduction in the long-term GFR slope [211], but in contrast with the post-hoc analyses of the ONTARGET and the *Telmisartan Randomized Assessment Study in ACE Intolerant Subjects With Cardiovascular Disease* (TRANSCEND) trials [212].

In addition to the risk of acute deterioration of kidney function, RAS-blocking agents inhibit the action of aldosterone, which results in a greater tendency to hyperkalemia [213]. This can be potentially dangerous, especially in patients with markedly impaired GFR, with atherosclerosis, older adults, and patients taking other drugs or dietary supplements that may raise serum potassium concentration. Therefore, we included our suggestions for monitoring and dealing with these potential threats. As there are no controlled trials on this issue, our statement is an expert opinion only and follows the statements from the latest KDIGO recommendations for the treatment of hypertension and the management of hyperkalemia [140,214].

### 4.2. Statement 4.2

We recommend not using a combination of ARB and ACEi [1A].

- *Comment to Statement 4.2* 

Combination therapy with ACEi and ARB concerning kidney protection has been studied extensively for years [215]. Several studies have investigated dual RAS blockade in diabetic or mixed cohorts of C215KD patients and documented a more significant antiproteinuric effect of combined therapy with ACEi and ARB as compared to monotherapy with either drug group. Despite that, randomized controlled trials not only evidenced that dual therapy does not improve renal outcomes but also noticed that such a combination may carry an increased risk of serious complications such as hypotension, AKI, and hyperkalemia [216].

In the ONTARGET trial, 25,620 participants (including 9603 patients with T2D) were randomly assigned to ACEi, ramipril 10 mg daily (n = 8576), ARB, telmisartan 80 mg daily (n = 8542), or a combination of both drugs (n = 8502; median follow-up was 56 months), and renal function and proteinuria were measured. The number of events for the composite primary outcome, including dialysis, doubling of S_cr_, and death was similar for telmisartan and ramipril but was increased with combination therapy. The secondary renal outcome, dialysis or doubling of S_cr_, was also similar to telmisartan and ramipril and more frequent with combination therapy [143]. In 2013, *the Veterans Affairs Nephropathy in Diabetes* (VA NEPHRON-D) study, a prospective, randomized trial testing the efficacy of the combination of ACEi and ARB versus ARB alone in 1448 patients with T2D with albuminuria (>300 mg/g of creatinine) on the composite endpoint of reduction in eGFR, progression to ESRD or death, was stopped prematurely because of futility. In this study, patients treated with double RAS blockade more frequently developed AKI and significant hyperkalemia [217]. Given the above, we recommend not using a combination of ARB and ACEi. For the same reasons, the combination of ACEi or ARB with the renin inhibitor aliskiren is not recommended in T2D [218], despite numerous studies indicating that aliskiren can also effectively reduce albuminuria [217].

### 4.3. Statement 4.3

#### 4.3.1. Statement 4.3.1

We recommend using a combination of ARB or ACEi with non-steroidal MRA—finerenone in CKD patients with T2D and with eGFR ≥ 25 mL/min/1.73 m^2^, serum potassium concentration ≤ 4.8 mmol/L, and urinary albumin/creatinine ratio ≥ 30 mg/g [1A]. Such treatment should be carried out with frequent monitoring of serum potassium concentration [2D]. In the case of hyperkalemia, we suggest treatment as it was described in statement 3.6.

- *Comment to Statement 4.3.1* 

As we already discussed in this document, the steroidal MRA reduces BP in CKD patients with resistant hypertension [167]. It is also known that mineralocorticoid receptor activation propagates kidney injury: inflammation, fibrosis, and CKD progression [219]. Therefore, MRA may be a potentially attractive adjunct to nephroprotective therapy regardless of its hypotensive effect. Several studies demonstrated beneficial effects on urinary albumin excretion in diabetic CKD by adding spironolactone to ACEi or ARB therapy [220]. However, the potentially beneficial effects on renal outcomes were confounded by an increased risk of hyperkaliemia, a factor limiting the prescribing of the steroidal MRA in CKD [221]. This is why the widespread use of such treatment has not been adopted, and the beneficial effect of spironolactone on long-term renal outcomes has yet to be proven. As we already mentioned about BP lowering, the alternative agents to spironolactone are eplerenone and finerenone. Antiproteinuric properties of eplerenone were documented in patients with T2D [222], and importantly, such effects can be additive to SGLT2i, as shown in the ROTATE-3 study [223]. However, eplerenone is still registered for the treatment of heart failure only.

In response to concerns related to hyperkalemia, several new selective nonsteroidal MRAs have developed. The only nonsteroidal MRA for which long-term clinical outcomes have been rigorously ascertained is finerenone [224]. In a prespecified, pooled individual-level analysis from two randomized trials, the FIDELIO-DKD and the FIGARO-DKD, including over 13,000 participants, a reduction in kidney failure outcome with finerenone on top of standard care with RASi was evidenced in patients with T2D (eGFR > 25 mL/min/1.73 m^2^) and albuminuria [225]. In detail, it was shown a lower incidence of the kidney composite of kidney failure, >57% decrease in eGFR, or death from kidney causes among those treated with finerenone (HR: 0.77; 95% CI: 0.67–0.88), and a lower incidence of kidney failure, defined as the initiation of chronic dialysis or kidney transplantation (HR: 0.80; 95% CI: 0.64–0.99). Finerenone was started at a dose of 20 mg daily when eGFR was >60 mL/min/1.73 m^2^ or at a dose of 10 mg daily when eGFR was 25–59 mL/min/1.73 m^2^, with up-titration to 20 mg daily if serum potassium remained ≤4.8 mmol/L [225].

There are no studies to prove that the addition of finerenone to the combination therapy of RASi and SGLT-2i provides additional nephroprotection benefits. SGLT-2 inhibitors were not standard of nephroprotection when the FIDELIO-DKD and FIGARO-DKD trials were initiated. However, 4.6 and 8.4% of FIDELIO-DKD and FIGARO-DKD were using SGLT-2 inhibitors at baseline, and the beneficial renal effects of finerenone, compared with placebo, appeared to be at least as beneficial among people using versus not using SGLT-2 inhibitors [226]. This applies to both clinically relevant improvement in albuminuria and reduction of the risk of CKD progression. The special benefit of having SGLT-2 inhibitors in such a triple combination therapy (finerenone, RASi, and SGLT-2i) may also be a reduced risk of hyperkalemia [226].

As shown by the estimated calculations presented in the latest meta-analysis by Neuen and colleagues, the combined use of RASi, SGLT2i, finerenone, and semaglutide has the most beneficial effect on the renal outcome compared to 2- and 3-drug combinations of these agents [227]. The question remains in what order to combine these medications. This is due to the lack of controlled clinical trials that would compare different strategies and drug combinations. Therefore, a possible therapy strategy may be based only on the regimen used in pivotal studies on individual groups of drugs and their secondary analyses and may only be treated as an expert opinion. The meta-analysis of Neuen et al. seems to be helpful in this respect as well, as it estimates the strength of the impact of individual agents and their combinations on the renal outcome [227].

Therefore, it is proposed that treatment should be started with RASi titrating to a maximally tolerated dose before introducing other medications, as was conducted in pivotal clinical trials. Taking into account the estimated strength of the nephroprotective effect of remaining drugs, we suggest adding SGLT2i secondly, and if the urinary albumin/creatinine ratio persists at ≥30 mg/g, finerenone should be further stepwise administered. Given that most of these drugs may affect intrarenal hemodynamics and cause an acute decrease in eGFR immediately after starting treatment, it is not recommended to introduce them at the same time; subsequent medications should be added stepwise with a minimum interval of one week to allow for equilibration of kidney function. During the chronic phase of treatment—to maintain the patients’ compliance—a single pill combination should be considered if in the future available—similarly to what is currently recommended in the treatment of hypertension.

#### 4.3.2. Statement 4.3.2

Concomitant use of spironolactone and non-steroidal MRA—finerenone is contraindicated [expert opinion]. In patients already treated with finerenone with resistant hypertension, we suggest replacing finerenone with spironolactone [expert opinion].

- *Comment to Statement 4.3.2* 

There are no premises or evidence indicating the validity of the combined use of steroid and non-steroid MRA. This combination may result in life-threatening hyperkalemia. Please note that finerenone is currently only approved for patients with T2D and albuminuria. Therefore, if it is necessary to use MRA for other indications, e.g., heart failure, hyperaldosteronism, or resistant hypertension, agents whose effectiveness has been confirmed in these diseases, e.g., spironolactone or eplerenone, should be used. Also, in patients who received finerenone for nephroprotection and who require MRA for indications other than nephroprotection, finerenone should be replaced with the steroid MRA with proven effectiveness in this regard. As there are no studies on this issue, our statement is an expert opinion only and follows the statements from the latest KDIGO 2022 Clinical Practice Guideline for Diabetes Management in Chronic Kidney Disease [32].

## 5. Metabolic Acidosis Treatment

### 5.1. Statement 5.1

We recommend measuring venous serum or blood bicarbonate concentration [HCO_3_^−^] in all CKD patients with T2D. Metabolic acidosis should be diagnosed when the venous serum or blood [HCO_3_^−^] is lower than 22 mmol/L [2B].

- *Comment to Statement 5.1* 

In our opinion, the range of screened CKD populations, diagnostic procedures, and the threshold diagnostic value for metabolic acidosis (i.e., non-respiratory acidosis) formulated for non-diabetic CKD patients should also apply to patients with CKD with T2D [1,173].

The results of the number of clinical studies showed that metabolic acidosis is frequent in CKD patients (for review—see [228]), and the presence of diabetes in these patients does not influence the prevalence of metabolic acidosis [229,230,231,232,233,234]. In the *Chronic Renal Insufficiency Cohort* (CRIC) study, which involved 3939 patients with CKD stages 2–4, metabolic acidosis was found in 17% of patients [229]. In this study, the frequency of metabolic acidosis was similar in diabetic and non-diabetic CKD patients [229]. In another study with 500 patients with CKD stages 1–5, it was shown that metabolic acidosis occurred in 20% of patients, and there were also no differences in the frequency of metabolic acidosis between CKD patients with and without diabetes [230]. This observation was further confirmed by Kuczera et al. [231]. In their cross-sectional study, there was no difference in the frequency of metabolic acidosis between patients with different causes of CKD (metabolic acidosis occurred in 25% of CKD patients with diabetes) [231]. Tangri et al., in the retrospective observational analysis of 136,067 CKD stages 3–5 patients (for stage 5—not yet on dialysis), did not find that diabetes influences the risk of metabolic acidosis [232].

The prospective observational CRIC study involved 3904 patients (48.5% of them with diabetes) and documented that metabolic acidosis contributes to CKD progression [220]. In this study, CKD progression risk (i.e., eGFR reduction by ≥50% or kidney replacement therapy initiation) in patients with serum bicarbonate concentration < 22 mmol/L was about three times higher than in patients with serum bicarbonate concentration > 26 mmol/L [233]. There was no interaction of CKD progression attributed to metabolic acidosis with diabetes [233]. The results of a retrospective observational study in CKD patients (including diabetics) documented that in patients with serum bicarbonate concentration < 22 mmol/L, mortality is increased when compared with CKD patients with higher serum bicarbonate [233]. Considering the above-quoted studies showing the high frequency of metabolic acidosis in CKD patients and its unfavorable clinical consequences, we recommend measuring venous serum or venous blood bicarbonate concentration in all CKD patients with T2D.

The threshold value of 22 mmol/L for diagnosing metabolic acidosis in CKD was established based on the above-quoted observational studies showing that CKD patients with plasma or venous blood bicarbonate concentrations below 22 mmol/L are characterized by increased CKD progression and higher mortality [235,236]. In the above-cited studies, T2D patients also participated, and no interaction of CKD progression related to acidosis with diabetes was found. Therefore, in our opinion, the threshold diagnostic value of metabolic acidosis should be the same among CKD patients with and without T2D.

### 5.2. Statement 5.2

We suggest the administration of sodium bicarbonate in CKD patients with TD2 and metabolic acidosis to prevent CKD progression to achieve venous serum or venous blood [HCO_3_^−^] in the range of 24–28 mmol/L [2B].

- *Comment to Statement 5.2* 

Since the interventional studies on the treatment of metabolic acidosis with sodium bicarbonate embraced T2D patients, in our opinion, sodium bicarbonate should be used in the same way, independently from the DM status [1]. The treatment target value of serum or blood bicarbonate concentration (i.e., 24–28 mmol/L) among CKD patients with and without T2D should also be the same.

To date, the largest prospective clinical study on treating metabolic acidosis in CKD patients was the *Use of Bicarbonate in Chronic Renal Insufficiency Study* (UBI)) [222]. In this study, 740 CKD patients with eGFR < 60 mL/min/1.73 m^2^ and a serum bicarbonate concentration of 18–24 mmol/L were randomly assigned to sodium bicarbonate therapy versus no study medication. In this study, 317% of participants suffered from diabetes. The treatment aimed to achieve a serum bicarbonate concentration in the range of 24–28 mmol/L. The oral dose of sodium bicarbonate used to achieve this goal was about 6 g daily. The observation period was 30 months. In patients undergoing sodium bicarbonate treatment, the annual reduction in eGFR was less than in those with no study medication (1.4 vs. 3.4 mL/min/1.73 m^2^, *p* < 0.0001). Seven percent of patients using sodium bicarbonate initiated dialysis, compared to 12% with no study medication (*p* = 0.02). Moreover, the mortality of patients receiving sodium bicarbonate was lower than in those who remained untreated (3% vs. 7%; *p* = 0.005) [235]. The results of a UBI study indicate that the target value for serum or blood bicarbonate should be set at 24–28 mmol/L [236].

Hultin et al., in a meta-analysis of 15 clinical trials (2 trials included only participants with diabetes mellitus, whereas two trials excluded participants with diabetes mellitus), with 2445 participants and a median follow-up of 12 months, showed that sodium bicarbonate therapy compared with placebo or no study medication slowed down the decline of eGFR (SMD: 0.26; 95% CI, 0.13–0.40) and reduced the risk of renal replacement therapy initiation (RR: 0.53; 95% CI, 0.32–0.89) [236]. Moreover, the results of the above-quoted interventional studies documented that treating CKD patients with sodium bicarbonate is safe [235,236].

A typical Western diet with meat and a low amount of fresh fruits and vegetables is acidifying and may worsen CKD-related metabolic acidosis CKD [237,238]. In small randomized clinical studies in CKD patients, it was documented that a diet rich in vegetables and fruits prescribed by a dietician and delivered to patients’ homes free of charge corrected metabolic acidosis [239,240,241]. However, these interventional studies excluded CKD patients with diabetes, mainly because of the potential risk of hyperkalemia. Therefore, we do not recommend such a diet as a routine method of treatment of metabolic acidosis in CKD patients with T2D.

## 6. Limitations of Current Evidence Concerning Pharmacological Nephroprotection

Although, in our opinion, the newer drugs introduced recently to the therapy of T2D and DKD, i.e., SGLT2i, GLP1RA, and non-steroidal MRAs (now solely represented by finerenone), have excellent and pivotal scientific documentation for their use; still some knowledge gaps could be identified.

The first and obvious area of future research is the utility of all drug classes in kidney transplant recipients. The bulk of evidence exists on the utility of both SGLT2i and GLP1RA in renal transplant recipients (recently reviewed by Bellos et al. and Clemens et al.) [242,243]. Nevertheless, these trials are limited to non-interventional observations, in some instances using the propensity match approach. Although the overall sound of these observations is positive in terms of improved metabolic control of diabetes, prevention of graft loss, and CV morbidity, randomized controlled trials are needed in this area. The use of finerenone and older MRAs in renal transplant recipients is well justified, especially given the fact that CV morbidity remains the leading cause of death in this patient group (most of them die with functioning grafts). The strong pathophysiological background for antagonizing mineralocorticosteroid receptors was, however, not yet translated into clinical observations or trials [244]. Finerenone may potentially be a candidate drug for such a randomized trial to test its efficacy in improving CV and renal (graft) outcomes in this patient group.

Although none of the drugs can protect kidneys in T2D patients on dialysis (although some of them may exert protection of so-called residual renal function [69]), there is still a potential to use them for cardiac protection. The very high mortality of patients on chronic dialysis on the one hand and the lack of any ‘positive’ clinical trial that would show the survival benefit of any cardioprotective drug on the other make testing SGLT2i or GLP1RA in this patient group especially tempting. We believe that in the case of both drug groups, patients would rather be randomized to ‘continuation’ and ‘discontinuation’ groups (as happened for ACEi and ARBs in the STOP-ACEi trial). An increasing number of observational data suggest that continuing rather than stopping SGLT2i upon dialysis commencement is safe and may be beneficial for future outcomes. GLP1RA use following dialysis start (especially long-lasting agents, such as semaglutide and dulaglutide) is less restricted as compared to SGLT2i, even in the guidelines, but the publications in this issue are scarce. As for today, finerenone use in dialysis patients seems questionable.

In our opinion, heart failure trials performed with SGLT2i do not adequately address the outcome of patients with CKD and diabetes. Except for the EMPEROR-Reduced trial, which analyzed CV outcome within several baseline ranges of UACR and eGFR, the remaining trials, i.e., EMPEROR-Preserved, DAPA-HF, and DELIVER, reported results only for patients with eGFR of less than 60 and above 60 mL/min/1.73 m^2^. Analyzing these seminal trials, it is hard to conclude if patients with HF, DKD, reduced eGFR, and with or without albuminuria also benefit from using SGLT2i [53,54,55,56,57,58].

## 7. Cost-Effectiveness of Pharmacological Nephroprotection

Two categories of drugs used for the treatment and prevention of diabetes and CKD in T2D patients, SGLT2i and GLP1RA, remain quite expensive, and their reimbursement differs depending on the country and type of health insurance system. The same holds for the one-in-the-class non-steroidal MRA, finerenone. Therefore, it is important to evaluate their cost-effectiveness in treating T2D. A recent meta-analysis on this topic concluded that all three SGLT2i available worldwide, i.e., canagliflozin, dapagliflozin, and empagliflozin, are cost-effective in the treatment of T2D diabetes and DKD, when added to the standard of care in high-income countries. The authors pointed out the lack of sufficient data from lower middle- and low-income countries to analyze their cost-effectiveness. It is worth mentioning that none of the studies included in this meta-analysis met the criteria of a high-quality cost-effectiveness evaluation study [245]. Several analyses performed in different countries aimed to address this problem. For example, in Japan, SGLT2i are cost-effective, especially in patients younger than 70 years of age, whereas such an effectiveness might be questioned in those older than 70 [246]. In countries with less privileged economic situations, this might not necessarily be true. For example, data from Thailand (based on patients with T2D and concomitant HF) indicate that QUALYs and outcomes significantly improve when SGLT2i are added to the standard of care, but cost-effectiveness remains questionable when taking into account the current prices of these drugs [247]. McEwan et al. analyzed the projected cost-effectiveness of dapagliflozin used in CKD in patients with and without diabetes, including the DAPA-CKD and DAPA-TIMI 58 trials, and reported the results separately for the UK, Spain, and Japan. The study found that QUALY gain obtained by adding the drug to the standard of care was cost-effective in all mentioned countries irrespective of diabetes status and UACR range [248]. The cost-effectiveness of SGLT2i in T2D (including DKD patients) was also predicted for the Netherlands and the UK [249,250]. One of the US-based analyses indicates that the present cost of drugs should be lowered by ~70% to become cost-effective in this country, reducing the daily cost of treatment with SGLTi or GLP1 to USD 5–6$ per day [251].

Regarding GLP1RA, the literature on cost-effectiveness is limited to a handful of publications. Taiwanese authors concluded, based on their analysis, that GLP1RA may be more cost-effective when calculating the cost per QALY gained as compared to long-acting insulin. Of interest, this analysis based on Taiwan’s National Health Insurance Research Database or published literature on the Taiwanese T2D population indicated that cost-savings from using GLP1RA can be expected, especially in T2D patients with CVD or CKD. The cost-effectiveness of GLP1RA vs. insulin therapy increases along with insulin therapy intensification [252,253]. GLP1RA was identified as more cost-effective even when compared to sulphonylureas when used as a second-line drug, given the striking reduction of diabetes-related complications in a population-based cohort study from Italy [254]. The data on economic aspects of using GLP1RA are almost entirely limited to the treatment of T2D itself, but not DKD. The appearance of new analyses on this topic should be expected following the successful semaglutide story in the FLOW trial.

It is worth mentioning a very complex analysis of newer drugs used to treat diabetes, which analyzed between-class comparisons and within-class comparisons of drugs in terms of their cost-effectiveness. The study found that cost-effectiveness may depend on the region analyzed, i.e., SGLT2i are more cost-effective as compared with GLP1RA in Asia, GLP1RA seems to be most effective in Europe, whereas the drug classes are equal in this regard in North America. Interestingly, empagliflozin and semaglutide (both injected and oral formulas) were found to be the most cost-effective ones in their respective classes of drugs. The authors point out the several limitations of the trials included in the analysis (i.e., high scientific quality but data insufficient to perform high-quality cost-effectiveness analyses). Many trials were also sponsored by drug manufacturers, and company employees were sometimes among the manuscript authors. Given the perspective of our statement, this important study did not analyze separately patients with CKD and T2D, but rather T2D patients in general [255]. Another meta-analysis found that both SGLT2i and GLP1RA are more cost-effective than sulphonylureas as the second-line treatment of T2D was not well controlled with metformin, though the comparison between SGLT2i and GLP1RA pointed to SGLT2i as more cost-effective than GLP1RA [256].

Analyzing the cost-effectiveness of finerenone in the treatment of DKD is easier given the registered indication of this drug, which is limited to the treatment of DKD. Some studies indicated the cost-effectiveness of finerenone added to the standard of care. Only a few papers were published in this field, indicating significant savings expected from risk reduction of CKD progression associated with the use of finerenone as an add-on to the standard of care in such countries as China, the Netherlands, and even in the United States, though price reductions would increase the utilization of a drug and improve cost-effectiveness [257,258].

To summarize, access to all mentioned drug groups may be limited in several countries, depending on economic status and healthcare system, with likely cost-effectiveness of therapy in high-income countries. In our opinion, the issue of SGLT2 and GLP1RA effectiveness is a ‘moving target’: negotiations between health authorities and manufacturers, discount systems, changing reimbursement criteria, etc., would steadily increase the availability of discussed drugs and improve cost-effectiveness. From the Polish perspective, patients with T2D and DKD have little or no limitations in access to all three SGLT2i available, whereas GLP1RA and finerenone remain very expensive when the present reimbursement criteria are applied.

## 8. Conclusive Remarks

The position paper underscores the value of implementing the recommendations as a universal, structured approach to nephroprotection in CKD with T2D, likely improving both renal and cardiovascular outcomes. This guidance reflects current advancements in pharmacological options, supporting clinicians in managing this high-risk population.

## 9. Brief Summary

**Figure 2:**
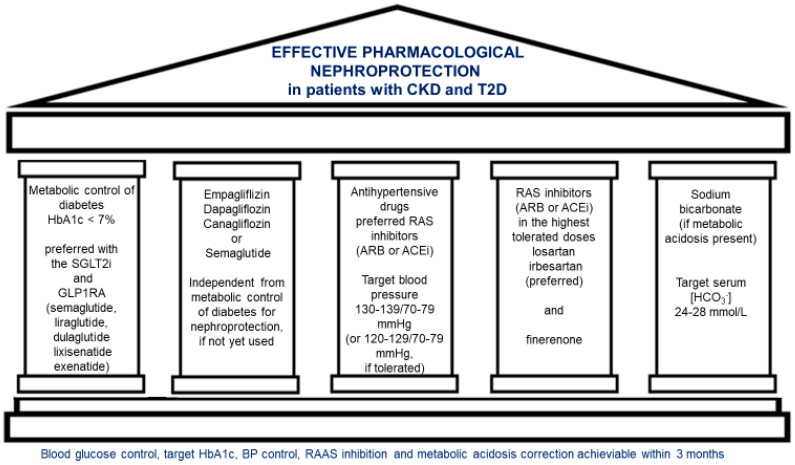
Five pillars of pharmacological nephroprotection in patients with chronic kidney disease and type 2 diabetes mellitus. Abbreviations: HbA1c—blood glycated hemoglobin concentration; SGLT2i—sodium–glucose transporter 2 inhibitors; GLP1RA—glucagon-like peptide 1 receptor agonist;ARB: angiotensin receptor blockers; ACEi: angiotensin-converting enzyme inhibitors.

In recent years, advancements in pharmacological nephroprotection have led to improved clinical guidelines for managing chronic kidney disease (CKD) in patients with type 2 diabetes mellitus (T2D). The current position statement by the Polish Society of Nephrology outlines a multifaceted nephroprotective strategy, which encompasses lifestyle modifications (healthy dietary patterns, regular physical exercise, smoking cessation, body mass optimization) and five key pillars (Table 3, Figure 2):

1. Effective Antihyperglycemic Treatment: Optimized glucose control is crucial, with sodium–glucose cotransporter-2 inhibitors (SGLT2i) or glucagon-like peptide-1 receptor agonists (GLP1RA) preferred for their nephroprotective or lowering albuminuria effects.
2. SGLT2 inhibitors and semaglutide: Independently from diabetic metabolic control, the use of SGLT2 inhibitors like empagliflozin, dapagliflozin, and canagliflozin or GLP1RA semaglutide is strongly recommended for their dual role in kidney protection and cardiovascular benefit, with a well-documented safety profile across CKD stages in T2D patients.
3. Antihypertensive Therapy: Optimal blood pressure management is essential, typically incorporating renin–angiotensin system inhibitors (RASi), specifically angiotensin receptor blockers (ARB) or, in the case of ARB intolerance, ACE inhibitors (ACEi), at the maximally tolerated dose.
4. RASi and Mineralocorticoid Receptor Antagonists: For T2D patients with persistent albuminuria, despite optimal use of ARB or ACEi and SGLT2i or semaglutide, finerenone, a non-steroidal mineralocorticoid receptor antagonist, is recommended to reduce kidney disease progression further.
5. Sodium Bicarbonate for Metabolic Acidosis: In patients with metabolic acidosis, sodium bicarbonate should be used with a target serum bicarbonate level of 24–28 mmol/L.

Notes for Clinical Practice

1. Monitoring: Regularly monitor renal function (eGFR, serum creatinine) and electrolytes (serum potassium and sodium) to manage the safety and efficacy of therapy.
2. Lifestyle Modifications: Encourage dietary adjustments, physical activity, smoking cessation, and weight management.
3. Individualization: Tailor treatment goals based on comorbidities, patient age, and risk factors.

## Figures and Tables

**Table 1:** Inclusion baseline HbA1c range and antihyperglycemic treatment in selected SGLT2i and GLP1RA trials with renal outcomes reported.

Trial Acronym	HbA1c Range at Baseline (Inclusion Criterion)	Antihyperglycemic Treatment			
		Active Arm		Placebo Arm	
CANVAS	7–10.5%	Insulin: 49.9% Metformin: 76.7% SU: 43.6% DPP4i: 12.0% GLP1RA: 3.8%		Insulin: 50.7% Metformin: 77.7% SU: 42.2% DPP4i: 13.0% GLP1RA: 4.3%	
EMPAREG-Outcome	7.0–9.0%	Insulin: 11.5% Metformin: 4.8% SU: 7.0% DPP4i: 8.3% GLP1RA: 2.4% TZD: 2.9%		Insulin: 5.8% Metformin: 3.7% SU: 3.8% DPP4i: 5.6% GLP1RA: 1.4% TZD: 1.2%	
DECLARE-TIMI 58	6.5–12.0%	Insulin: 41.6% Metformin: 81.8% SU: 42.1% DPP4i: 16.5% GLP1RA: 4.6%		Insulin: 40.2% Metformin: 82.2% SU: 43.2% DPP4i: 17.1% GLP1RA: 4.1%	
CREDENCE	6.5–12.0% (6.5–10.5% in Germany)	Insulin: 66.0% Metformin: 58.0% SU: 27.8% DPP4i: 17.2% GLP1RA: 4.0% TZD: 3.2% Alpha-glucosidase inhibitors: 3.0%		Insulin: 65.1% Metformin: 57.7% SU: 29.9% DPP4i: 17.0% GLP1RA: 4.3% TZD: 3.0% Alpha-glucosidase inhibitors: 3.3%	
LEADER	≥7.0%	Insulin: 43.7% Metformin: 75.8% SU: 50.8% DPP4i: <0.1% GLP1RA: 0 TZD: 6.3% Alpha-glucosidase inhibitors: 3.0% Glinides: 3.8		Insulin: 45.6% Metformin: 77.1% SU: 50.6% DPP4i: <0.1% GLP1RA: 0 TZD: 6.0% Alpha-glucosidase inhibitors: 2.6% Glinides: 3.7	
REWIND	≤9.5%	Insulin: 24.0% Metformin: 81.3% SU: 45.9% DPP4i: 5.4% TZD: 2.0% Other: 0.3%		Insulin: 23.7% Metformin: 81.1% SU: 46.1% DPP4i: 6.0% TZD: 1.4% Other: 0.4%	
PIONEER-6	No HbA_1c_ criterion	Insulin: 60.8% Metformin: 76.7% SU: 32.5% DPP4i: 0.1% GLP1RA: 0.1% TZD: 4.1% Alpha-glucosidase inhibitors: 2.3% SGLT2i: 10.4%		Insulin: 60.4% Metformin: 78.0% SU: 32.0% DPP4i: 0 GLP1RA: 0 TZD: 3.3% Alpha-glucosidase inhibitors: 2.7% SGLT2i: 8.8%	
SUSTAIN-6	≥7.0%	Semaglutide 0.5 mg	Semaglutide 1.0 mg	Placebo 0.5 mg	Placebo 1.0
		Insulin: 58.0% Metformin: 74.7% SU: 42.3% DPP4i: 0.1% GLP1RA: 0.1% TZD: 1.7% Glinides: 3.0 SGLT2i: 0 Alpha-glucosidase inhibitors: 1.1%	Insulin: 58.0% Metformin: 72.3% SU: 42.5% DPP4i: 0.2% TZD: 2.6% Glinides: 2.8% SGLT2i: 0.1% Alpha-glucosidase inhibitors: 0.9	Insulin: 58.1% Metformin: 71.1% SU: 44.1% DPP4i: 0.2% TZD: 2.2% Glinides: 2.9% Alpha-glucosidase inhibitors: 1.9% SGLT2i: 0.2%	Insulin: 58.0% Metformin: 74.8% SU: 42.3% DPP4i: 0 TZD: 2.8% Alpha-glucosidase inhibitors: 2.8% SGLT2i: 0.2%

Footnotes: GLP1RA = glucagon-like peptide 1 agonists; SU = sulphonylurea; DPP4i = dipeptidyl peptidase 4 inhibitors; TZD = thiazolidinediones; in some trials, the data for biguanides were provided instead for metformin alone.

**Table 2:** Glucose-lowering therapies: current licensing based on estimated glomerular filtration rate (eGFR) [94,95,96].

		Renal Impairment-CKD Stage and eGFR (mL/min/1.73 m^2^)							
		G1–2	G3a	G3b	G4			G5	
Drug	Class of drug	eGFR > 60	eGFR 45–59	eGFR 30–44	eGFR 15–30			eGFR < 15	
**Metformin**	Biguanide			Reduce dose to 2 × 500 mg					
**Gliquidone**	Sulfonylurea								
**Glimepiride**	Sulfonylurea		Dose adjusted to glycemia, monitor CBG						
**Glipizide**	Sulfonylurea		Dose adjuste to glycemia, monitor CBG						
**Gliclazide**	Sulfonylurea		Dose adjusted to glycemia, monitor CBG						
**Repaglinide**	Meglitinide				Dose reduction advised, monitor CBG				
**Sitagliptin**	DPP4i			Reduce dose to 50 mg	Reduce dose to 25 mg				
**Saxagliptin**	DPP4i		Reduce dose to 2.5 mg, avoid treatment in dialyzed patients						
**Vildagliptin**	DPP4i		Reduce dose to 50 mg if eGFR < 50						
**Linagliptin**	DDP4i								
**Pioglitazone**	Thiazolidinediones								Avoid treatment in dialyzed patients
**Dulaglutide**	GLP1RA								
**Exenatide** **(twice daily)**	GLP1RA		Gradually increase dose if eGFR 30–50						
**Exenatide** **(once daily)**	GLP1RA								
**Liraglutide**	GLP1RA								
**Lixisenatide**	GLP1RA								
**Semaglutide** **(oral/injectable)**	GLP1RA								
**Tirzepatide**	GLP-1 and GIP agonist								Caution advised
**Dapagliflozin**	SGLT2i					If eGFR < 25 do not start, continuation only, discontinuation in dialyzed patients			
**Empagliflozin**	SGLT2i						If eGFR < 20 do not start, continuation only, discontinuation in dialyzed patients		
**Canagliflozin**	SGLT2i	Initiate at 100 mg and gradually increase to 300 mg if needed	Initiate at 100 mg or continuation		Do not start, continuation 100 mg, discontinuation in dialyzed patients				
**Acarbose**	Alpha-glucosidase inhibitor								

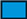
 no need to adjust the dose to eGFR. 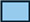 recommended dose adjustment to eGFR. 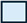 not recommended the use of the drug. Abbreviations: CBG, capillary blood glucose; CKD, chronic kidney disease; eGFR, an estimate of glomerular filtration rate, usually calculated using Cockcroft–Gault equation; DPP4i, dipeptidyl peptidase-4 inhibitors; GLP1RA = glucagon-like peptide 1 ago-nists; GIP, glucose-dependent insulinotropic polypeptide; SGLT2i, sodium–glucose co-transporter-2 inhibitor.

**Table 3:** Nephroprotection Recommendations in CKD and T2D patients. Summary Table.

Pillar/ Level of Recommendation	Recommendations	Key Drugs
1. Effective Antihyperglycemic Treatment (1A)	Maintain HbA1c <7% to prevent CKD progression. Adjust targets (7–8%) in patients at high hypoglycemia risk. Caution glucose monitoring in advanced CKD. Antihyperglycemic drugs needed to be chosen based on eGFR with dose adjustment and treatment cessation according to eGFR. Use SGLT2 inhibitors. For advanced CKD, consider GLP-1 receptor agonists.	- SGLT2i: empagliflozin, dapagliflozin, canagliflozin - GLP1RA: semaglutide, dulaglutide, liraglutide
2. Sodium–Glucose Co-Transporter 2 Inhibitors (SGLT2i) and Semaglutide (1A)	Initiate SGLT2i or semaglutide for T2D and CKD, except when eGFR is below drug-specific thresholds. Continue even in advanced CKD stages if tolerated, until dialysis or transplant.	- Empagliflozin - Dapagliflozin - Canagliflozin - Semaglutide
3. Blood Pressure Control (1B)	Tailor antihypertensive treatment to reach individualized blood pressure targets: 130–139/70–79 mmHg or 120–129/70–79 mmHg if tolerated. Use RASi (ARB/ACEi) in the first line at the maximally tolerated doses.	- ARBs: e.g., losartan, irbesartan
4. Renin–Angiotensin System Inhibition (RASi) (1A)	Use angiotensin II receptor blockers (ARBs) or, in the case of ARBs, intolerance ACE inhibitors (ACEi) as first-line nephroprotective agents. Combine with SGLT2i or GLP1RA when possible. Add finerenone in cases of persistent albuminuria (>30 mg/day) despite RAS blockade and SGLT2i use if hyperkalemia risk is low.	- ARBs: e.g., losartan, irbesartan - Finerenone
5. Management of Metabolic Acidosis (2B)	Treat with sodium bicarbonate to maintain serum bicarbonate levels within the range 24–28 mmol/L.	- Sodium bicarbonate

## Data Availability

Not applicable.
